# 17α-Ethinyl estradiol-3-sulfate increases survival and hemodynamic functioning in a large animal model of combined traumatic brain injury and hemorrhagic shock: a randomized control trial

**DOI:** 10.1186/s13054-021-03844-7

**Published:** 2021-12-16

**Authors:** Andrew R. Mayer, Andrew B. Dodd, Julie G. Rannou-Latella, David D. Stephenson, Rebecca J. Dodd, Josef M. Ling, Carissa J. Mehos, Cidney R. Robertson-Benta, Sharvani Pabbathi Reddy, Rachel E. Kinsler, Meghan S. Vermillion, Andrew P. Gigliotti, Veronik Sicard, Amy L. Lloyd, Erik B. Erhardt, Jessica M. Gill, Chen Lai, Vivian A. Guedes, Irshad H. Chaudry

**Affiliations:** 1 The Mind Research Network/Lovelace Biomedical Research Institute, Pete & Nancy Domenici Hall, 1101 Yale Blvd. NE, Albuquerque, NM 87106 USA; 2 Neurology Department, University of New Mexico School of Medicine, Albuquerque, NM 87131 USA; 3 Psychiatry Department, University of New Mexico School of Medicine, Albuquerque, NM 87131 USA; 4 Psychology Department, University of New Mexico, Albuquerque, NM 87131 USA; 5 Neurosciences Department, University of New Mexico School of Medicine, Albuquerque, NM 87131 USA; 6 Department of the Army Civilian, U.S. Army Aeromedical Research Laboratory, Fort Rucker, AL 36362-0577 USA; 7 Goldbelt Frontier, Inc., U.S. Army Aeromedical Research Laboratory, Fort Rucker, AL USA; 8 Department of Mathematics and Statistics, University of New Mexico, Albuquerque, NM 87131 USA; 9 National Institute of Nursing Research, National Institutes of Health, Bethesda, MD 20892 USA; 10 Department of Surgery, University of Alabama at Birmingham, Birmingham, AL 35294-0019 USA

**Keywords:** Hypovolemia, Brain injuries, Traumatic, Estrogens, Swine, Hemodynamics, Multiple trauma

## Abstract

**Background:**

Traumatic brain injury (TBI) and severe blood loss resulting in hemorrhagic shock (HS) represent leading causes of trauma-induced mortality, especially when co-occurring in pre-hospital settings where standard therapies are not readily available. The primary objective of this study was to determine if 17α-ethinyl estradiol-3-sulfate (EE-3-SO_4_) increases survival, promotes more rapid cardiovascular recovery, or confers neuroprotection relative to Placebo following TBI + HS.

**Methods:**

All methods were approved by required regulatory agencies prior to study initiation. In this fully randomized, blinded preclinical study, eighty (50% females) sexually mature (190.64 ± 21.04 days old; 28.18 ± 2.72 kg) Yucatan swine were used. Sixty-eight animals received a closed-head, accelerative TBI followed by removal of approximately 40% of circulating blood volume. Animals were then intravenously administered EE-3-SO_4_ formulated in the vehicle at 5.0 mg/mL (dosed at 0.2 mL/kg) or Placebo (0.45% sodium chloride solution) via a continuous pump (0.2 mL/kg over 5 min). Twelve swine were included as uninjured Shams to further characterize model pathology and replicate previous findings. All animals were monitored for up to 5 h in the absence of any other life-saving measures (e.g., mechanical ventilation, fluid resuscitation).

**Results:**

A comparison of Placebo-treated relative to Sham animals indicated evidence of acidosis, decreased arterial pressure, increased heart rate, diffuse axonal injury and blood–brain barrier breach. The percentage of animals surviving to 295 min post-injury was significantly higher for the EE-3-SO_4_ (28/31; 90.3%) relative to Placebo (24/33; 72.7%) cohort. EE-3-SO_4_ also restored pulse pressure more rapidly post-drug administration, but did not confer any benefits in terms of shock index. Primary blood-based measurements of neuroinflammation and blood brain breach were also null, whereas secondary measurements of diffuse axonal injury suggested a more rapid return to baseline for the EE-3-SO_4_ group. Survival status was associated with biological sex (female > male), as well as evidence of increased acidosis and neurotrauma independent of EE-3-SO_4_ or Placebo administration.

**Conclusions:**

EE-3-SO_4_ is efficacious in promoting survival and more rapidly restoring cardiovascular homeostasis following polytraumatic injuries in pre-hospital environments (rural and military) in the absence of standard therapies. Poly-therapeutic approaches targeting additional mechanisms (increased hemostasis, oxygen-carrying capacity, etc.) should be considered in future studies.

**Supplementary Information:**

The online version contains supplementary material available at 10.1186/s13054-021-03844-7.

## Background

Traumatic brain injury (TBI) and severe blood loss resulting in hemorrhagic shock (HS) individually represent leading causes of trauma-induced mortality and are especially detrimental when combined [1–3]. Hypothermia, acidosis and coagulopathy represent the hallmark complications of HS, ultimately resulting in oxygen debt at the tissue level [4]. Hypovolemia also decreases arterial pressure and increases vasoconstriction, resulting in earlier and more severe cerebral dysautoregulation, reduced blood flow, hypoxia, increased contusion volume and a doubling of mortality rate in concurrent TBI + HS [5, 6]. Although fluid resuscitation is recommended for HS, unless carefully managed it can also exacerbate brain edema and elevate intracranial pressure [7]. Similarly, hyperventilation helps to restore systemic acid–base balance following HS [8], whereas respiratory depression is the most common cause of death in preclinical models of moderate-to-severe TBI [9]. Respiratory complications are also more common in TBI + HS relative to HS alone in humans [10]. Thus, the optimal resuscitation approaches for concurrent TBI + HS remain actively debated [3, 7].

Care is further complicated in remote settings (e.g., injuries occurring in the wilderness, developing countries, or military settings) where resuscitative fluids and blood products are not readily available [11, 12]. Death typically ensues in little more than one hour in the absence of intravenous fluid resuscitation in Class III or IV trauma patients [13]. The family of estrogens (17β-estradiol and 17α-ethinyl estradiol-3-sulfate [EE-3-SO_4_]) are naturally occurring steroid hormones that are beneficial in HS [14, 15] and have been shown to be neuroprotective across multiple neural injury models [16, 17]. Specifically, EE-3-SO_4_ has been shown to increase short-term survival rate (e.g., 3–6 h) in both rodent [18] and swine [15] models of HS in the absence of typical doses of resuscitation fluids. Proposed mechanisms of action for EE-3-SO_4_ include increased cardiac ejection fraction and vasodilation [19–22], increased mitochondrial respiratory complex activity in the myocardium [18, 23], increased cell survival pathways concomitant with decreased cell death pathways, as well as decreased metabolic acidosis and glucose derangement [15, 21]. All of the above effects are dependent on estrogen receptor engagement, where specificity was recently confirmed by estrogen receptor antagonists [20].

Estrogens freely cross the blood–brain barrier (BBB) and have been demonstrated to maintain and regulate the BBB in both humans and rodents [24, 25]. In terms of neuroprotection, seminal work suggests estrogens reduce lesion size and lessen the extent of cell death in the injured brain [17], as well as potentially promoting vascular regeneration following injury [26]. It has been suggested that the estrogen-mediated maintenance of the BBB may also reduce edema after stroke via dampening of the Na–K–Cl cotransporter mechanism [27]. Most pertinent to the current study, EE-3-SO_4_ administered one hour following TBI in rodent models resulted in a reduction in intracranial pressure, edema and neuroinflammation while increasing cerebral perfusion pressure and partial pressure of oxygen in brain tissue [17, 28–30], but did not affect markers of diffuse axonal injury [30].

Due to similar homology in terms of hemostatic mechanisms, cardiovascular systems and brain structure (gyrencephalic, similar gray-white matter ratios), swine represent the most commonly utilized species for large animal models of TBI + HS [3]. The majority of these studies have primarily utilized controlled cortical impact or fluid percussion injury, even though closed-head, acceleration injuries represent the most common form of human TBI [31]. A recent study reported acute mortality rates of approximately 88% and 13%, respectively, in an acceleration model of TBI with either 55% or 40% blood loss in the absence of any treatment relative to Shams [32]. In addition to traditional metrics of metabolic derangement associated with HS, results from this study also validated the sensitivity of several blood-based biomarkers for measuring diffuse axonal injury, blood–brain barrier breach and neuroinflammation in swine (glial fibrillary acidic protein [GFAP], neurofilament light chain [NFL], ubiquitin C-terminal hydrolase [UCH-L1], amyloid-beta 40 [Aβ40] and 42 [Aβ42]) that are commonly used in clinical settings [33]. To our knowledge, there have been no studies examining the efficacy of EE-3-SO_4_ in a large animal model of TBI + HS.

The current study therefore had two primary aims. The first was to attempt to replicate previous findings of metabolic derangement and neurotrauma in a swine model of closed-head, accelerative TBI + HS (i.e., Placebo-treated animals relative to uninjured Shams). The second aim examined the efficacy of EE-3-SO_4_ to prolong survival in a pre-hospital environment that mimicked more austere levels of care (absence of resuscitative fluids or mechanical ventilation; [32]). Based on previous literature [15, 21], we postulated that EE-3-SO_4_ would increase survival time and improve hemodynamic functioning, while subsequently decreasing markers of metabolic acidosis, BBB breach and neuroinflammation. Second, we also predicted that there would be a statistically null effect for blood-based and immunohistochemical markers of diffuse axonal injury [30].

## Methods

### Animal preparation

The methods used in the current study are nearly identical to a previous publication [32] and are therefore only briefly presented here. All animal procedures (see Table 1) were approved by the local Institutional Animal Care and Use Committee (Lovelace Biomedical, FY17-077, FY20-133) and the US Army Medical Research & Development Command Office of Research Protections Animal Care and Use Review Office (DM160115, DM160115.e001) prior to study initiation. The study was conducted in accordance with Animal Research: Reporting In Vivo Experiments 2.0 guidelines [34]. Specifically, eighty sexually mature Yucatan swine (28.18 ± 2.72 kg; 40 females and 40 males; 190.64 ± 21.04 days old at the time of experimental procedures) were obtained from Premier Biosource (formerly S&S Farms, Ramona, California, USA). Animals were screened and vaccinated for common swine diseases by the vendor prior to arrival at the research facility. Upon arrival, animals were examined by a veterinarian and underwent a quarantine (i.e., no experimental procedures) and acclimation period of 7 days, with observation daily prior to the start of experimental procedures. Animals were single-sex group-housed when possible (exceptions made for odd number of animals per sex or behavioral incompatibility) in indoor runs on a 12:12 light/dark cycle. Environmental conditions were maintained between ~ 16–27 °C and ~ 30–70% relative humidity. Animals were limit fed (based on age and weight) twice per day and had ad libitum access to water. Animals were randomly assigned in a blocked fashion to the experimental drug (EE-3-SO_4_ vs. Placebo) and the actual/mock rough ground transport conditions, or to the uninjured Sham cohort. The blocked assignment controlled for biological sex and experimental arm to alleviate concerns about potential time-related effects. All in-life procedures with the exception of rough ground transport, data quality assurance and data scoring were conducted in a blinded fashion, with blind broken immediately prior to conducting the final analyses. Rough ground transport did not significantly affect survival rates (*p* > 0.10) and therefore will be presented in a separate manuscript.

**Table 1:**
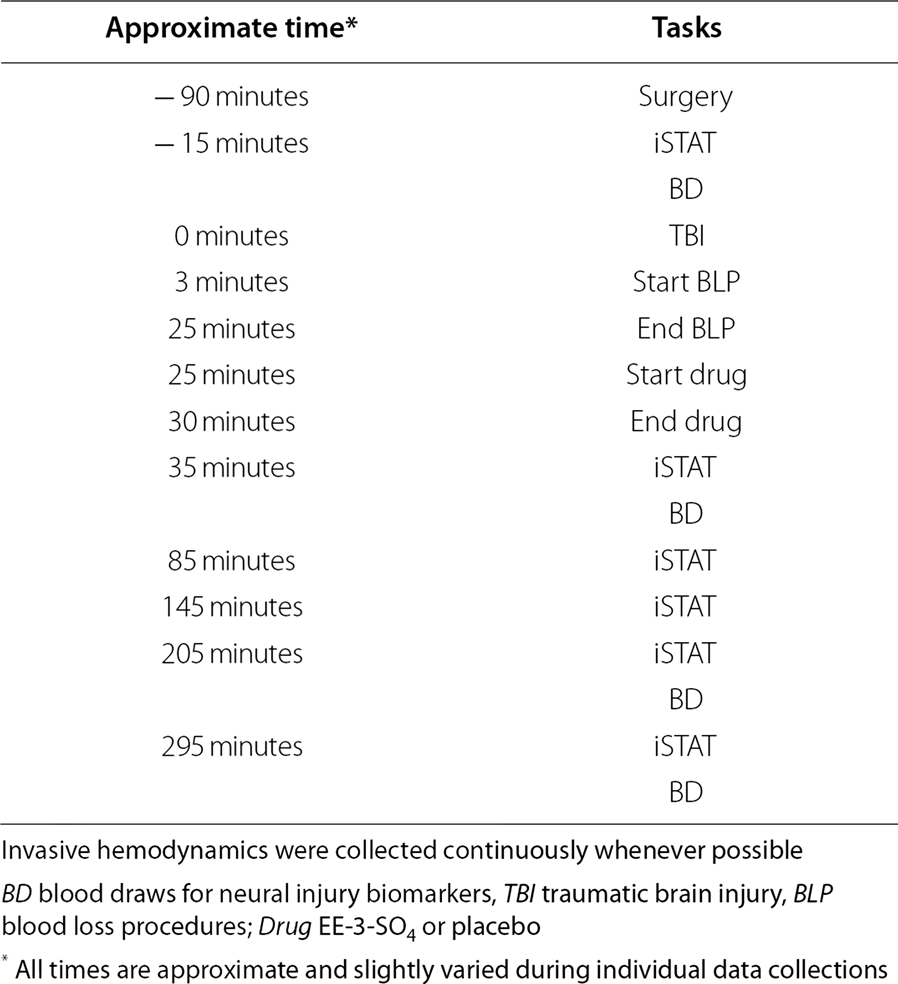
Timeline of critical experimental procedures

During experimental procedures, animals were maintained under general anesthesia using a combination of isoflurane, midazolam and ketamine (see Additional file 1 for dosing). Femoral arteries were catheterized and flushed every 20 min in conjunction with a single artificial breath. Blood samples were obtained and analyzed using point-of-care devices for primary (glucose, lactate and bicarbonate [HCO_3_]) and secondary (potential hydrogen [pH], partial CO_2_ pressure [PCO_2_], sodium [Na], potassium [K], ionized calcium [iCa]) variables. A Simoa HD-1 Analyzer was used to determine concentrations of NFL, UCH-L1 and GFAP (primary outcome measures), as well as Aβ40 and Aβ42 (secondary outcome measures). Invasive arterial pressure monitoring was used to calculate primary (shock index [SI]; pulse pressure [PP]) and secondary (mean arterial pressure [MAP] and heart rate) hemodynamic variables in 30-s epochs. Focused analyses examined the effects of EE-3-SO_4_ on hemodynamics at 5 min and 20 min immediately post-administration.

A closed-head TBI was initiated via a pneumatic device targeting a rotation of 250 radians/second in the coronal plane [32] with a subset of animals monitored for actual head kinematics [35]. Animals were immediately placed in lateral recumbency and subjected to arterial hemorrhage via controlled removal of approximately 40% of estimated total blood volume 161 ± 48.7 s after the TBI. Animals were then administered EE-3-SO_4_ (formulated in the vehicle at 5.0 mg/mL and dosed at 0.2 mL/kg) or Placebo (0.45% sodium chloride solution) as an intravenous administration over 5 min via a continuous pump (0.2 mL/kg) and monitored.

Single immunohistochemistry labeling was performed to examine for extravasated serum proteins (Immunoglobulin G; IgG) as markers of blood–brain barrier integrity, axonal pathology (amyloid precursor protein; APP), and upregulation of microglia (ionized calcium-binding adaptor molecule 1; IBA1).

### Statistical plan

The first series of analyses attempted to replicate previous observations [32] of blood-based biomarker findings of metabolic derangement and neurotrauma in a swine model of closed-head, accelerative TBI + HS (i.e., Placebo vs. Sham). All terminal samples from non-surviving animals were excluded from analyses due to extreme physiological derangement (e.g., 5 min of apnea) and non-standard data collection times. Generalized linear models (GLM) with appropriate (Gaussian or Gamma) response distributions determined by the model fit or linear mixed-effects (LME) models were utilized for analyses (see Additional file 1). Similar to a previous publication [32], baseline measurements were used as a covariate for all analyses when available.

The second series of analyses examined the efficacy of EE-3-SO_4_ in promoting survival and improving physiological endpoints (i.e., EE-3-SO_4_ vs. Placebo). Any animal that did not survive at least 20 min post-blood loss was excluded from drug-focused analyses. Survival rates between the cohorts were assessed using a Cox proportional-hazards model test. Similar tests (GLM, LME) and methodologies (e.g., baseline as a covariate, terminal samples excluded) were used to investigate differences in physiological markers between EE-3-SO_4_ and Placebo-treated animals. Any data that were collected with minor variations in protocol were individually reviewed for outlier status (see Additional file 1), with all analyses conducted with and without extreme outliers (results unchanged).

Finally, a series of LME models were fit to determine variables that differed between non-surviving and surviving animals independent of drug assignment. Specifically, surviving animals (*N* = 16) were matched to non-surviving animals (*N* = 16) based on biological sex, drug assignment and temporal cohort (whenever was possible). The two groups were compared across all primary and secondary variables based on the last successfully acquired timepoint prior to death (timepoint also matched to surviving animals), with matched data eliminated for blood-based biomarkers in the event of death occurring prior to acquisition. Both baseline measurements and acquisition time were entered as additional covariates into the model. The latter controlled for the fact that the temporal course of each biomarker was expected to fluctuate as a function of time post-injury [32]. Due to the exploratory nature of these analyses, individual tests were not corrected for multiple comparisons.

## Results

### Characterization of model pathology

No significant (all *p*’s > 0.05) group differences existed at baseline for hemodynamic, point-of-care, or neural biomarkers between Placebo (*N* = 34) and uninjured Sham (*N* = 12) cohorts. Significant Group × Time interactions were observed for glucose (*F*_4,35.93_ = 25.45, *p* < 0.001), lactate (*F*_4,22.21_ = 6.32, *p* = 0.001) and HCO_3_ (*F*_4,23.87_ = 7.03, *p* = 0.001) following Bonferroni correction (0.05/3 = 0.017; see Additional file 1: Fig. S1A). Follow-up tests indicated reduced HCO_3_ (all *p*’s < 0.001; Cohen’s *d* =  − 3.40 to − 1.68), increased glucose (all *p*’s < 0.001; *d* = 1.38 to 3.91) and increased lactate (all *p*’s < 0.001; *d* = 1.79 to 2.94) in the Placebo cohort, which all demonstrated evidence of an incomplete recovery trajectory at 295 min post-TBI. Secondary point-of-care (Bonferroni correction at 0.05/5 = 0.01) variables also demonstrated significant Group × Time interactions for Na (*p* < 0.001) and K (*p* < 0.001). Main effects of Group were observed for pH (*p* = 0.002), PCO_2_ (*p* = 0.004) and iCa (*p* < 0.001), with Time effects presented in Additional file 1.

Significant Group × Time interactions were also observed for the primary hemodynamic variables of PP (*F*_4,34.02_ = 41.50, *p* < 0.001) and SI (*F*_4,22.18_ = 10.72, *p* < 0.001) following Bonferroni correction (0.05/2 = 0.025). SI was significantly decreased post-TBI for the Placebo relative to Sham cohort (*p* = 0.002, *d* =  − 1.04), but then increased after blood loss with evidence of an incomplete recovery (all *p*’s ≤ 0.001; *d* = 1.56 to 1.89). In contrast, PP was significantly (*p*’s ≤ 0.003; *d* =  − 5.88 to − 1.11) reduced in Placebo animals immediately post-blood loss until 85 min post-TBI, with statistical evidence of full recovery at 145 min post-TBI (see Additional file 1: Fig. S1B). Significant Group × Time interactions (see Additional file 1) were also observed for secondary hemodynamic measurements of HR (*p* < 0.001) and MAP (*p* < 0.001).

Immunohistochemical results indicated significant increases in cortical APP (Wald-*χ*^2^ = 47.22, *p* < 0.001, *d* = 3.11), as well as cortical (Wald-*χ*^2^ = 7.01, *p* = 0.008, *d* = 1.14) and cerebellar (Wald-*χ*^2^ = 20.45, *p* < 0.001, *d* = 1.82) IgG following multiple comparison corrections (Bonferroni 0.05/4 = 0.013). Conversely, cortical IBA1 did not meet corrected significance (*p* = 0.082, *d* = 0.60), and this marker was excluded from further analyses. From a qualitative perspective, 24/34 animals in the Placebo cohort exhibited findings of intracranial bleeding on gross necropsy examination. This was most typically characterized by hemorrhage along the dorsal and ventral surfaces of the cerebellum, as well as the dorsal surface of the cortex in 7 animals. No Sham animals exhibited intracranial bleeding on gross necropsy examination.

For blood-based biomarkers (Additional file 1: Fig. S1C), significant Group × Time interactions were also observed for primary measures of NFL (*F*_1,32.00_ = 56.83, *p* < 0.001) and GFAP (*F*_1,32.85_ = 29.25, *p* < 0.001), with a main effect of Group (Placebo > Sham; *F*_1,34.30_ = 12.40, *p* = 0.001, *d* = 1.18) for UCH-L1 (Bonferroni correction 0.05/3 = 0.017). GFAP was significantly elevated across time points (both *p*’s < 0.001; *d* = 2.96 to 3.31) in the Placebo relative to Sham cohort, with a larger magnitude of difference at the terminal sample. In contrast, NFL was only significantly elevated in Placebo relative to Sham animals at the terminal sample (*p* < 0.001, *d* = 2.66). Secondary measures (see Additional file 1) of Aβ42 (*p* = 0.011) exhibited a significant Group × Time interaction while Aβ40 demonstrated an overall main effect of Group (*p* < 0.001; Placebo > Shams).

### Analyses testing efficacy of EE-3-SO_4_

A total of four animals expired either during the blood loss procedures (1 EE-3-SO_4_) or within 20 min of blood loss (2 EE-3-SO_4_; 1 Placebo) and were excluded from analyses based on a priori criteria. There were no statistical differences between remaining EE-3-SO_4_ (*N* = 31) and Placebo (*N* = 33) cohorts on age, body weight or anesthetic time for catheter placement (all *p*’s > 0.05). Comparison of HYGE parameters (Table 2) indicated no significant cohort effects for peak angular velocity, time-to-peak and deceleration time (all *p*’s > 0.05). Similarly, there were no significant differences between the groups in terms of total blood volume removed with the pump, or the amount of time that elapsed between the TBI and the onset of the blood loss procedure (all *p*’s > 0.05). Baseline ionized calcium (iCa) was significantly increased (uncorrected Wald-*χ*^2^ = 4.31, *p* = 0.038) for the EE-3-SO_4_ (1.41 ± 0.01 mmol/L) relative to Placebo (1.38 ± 0.01 mmol/L) cohort. Otherwise, there were no other significant group differences for all hemodynamic, point-of-care and neural biomarkers between EE-3-SO_4_ and Placebo groups at baseline (all *p*’s > 0.05; *d* = 0.00 to 0.52).

**Table 2:** Animal characteristics and HYGE parameters

	Placebo (*N* = 33)	EE-3-SO_4_ (*N* = 31)	*p*
Measure			
Weight (kg)	28.07 ± 3.22	28.12 ± 2.62	0.952
Age (days)	190.64 ± 22.57	186.65 ± 18.72	0.433
reTBV(%)	40.2 ± 1.4	40.2 ± 1.5	0.970
HYGE			
Peak velocity (rad/s)	249.25 ± 6.25	248.80 ± 6.35	0.777
Decel time (ms)	4.07 ± 4.04	3.61 ± 3.89	0.651
Time to peak (ms)	6.15 ± 0.24	6.15 ± 0.22	0.932

*Decel time* deceleration time, *ms* millisecond, *rad/s* radians per second, *reTBV* removed estimated total blood volume

**Fig. 1:**
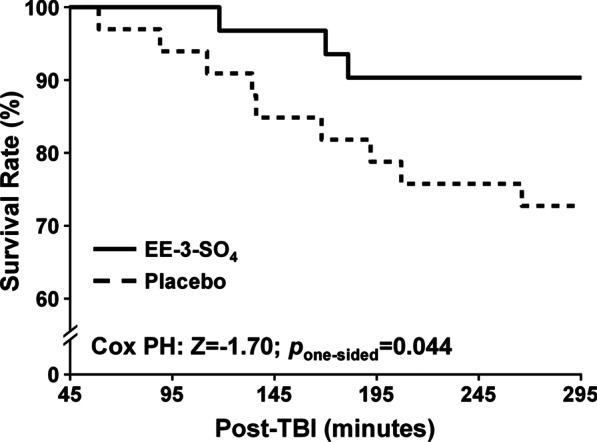
This figure depicts significantly increased survival rate and time (minutes post-traumatic brain injury [TBI]) for the EE-3-SO_4_ (solid line) relative to Placebo (dashed line) cohort based on a one-sided Cox proportional-hazards model (Cox PH). The end of the experiment occurred at 295 min post-TBI

The Cox proportional-hazards model indicated that the percentage of animals surviving to 295 min post-TBI (i.e., duration of the experiment; Fig. 1) was significantly higher (*β* =  − 1.14, *Z* =  − 1.70, *p*_one-sided_ = 0.044) for the EE-3-SO_4_ cohort (28/31; 90.3%) relative to Placebo cohort (24/33; 72.7%). Specifically, there were five deaths in the Placebo cohort relative to 1 death in the EE-3-SO_4_ cohort by 145 min following TBI, with an additional 4 deaths in the Placebo cohort and 2 deaths in the EE-3-SO_4_ cohort between 145 and 295 min. The majority of animals in both the Placebo (7/9) and EE-3-SO_4_ (3/3) groups expired as a result of respiratory arrest rather than cardiac arrest.

**Fig. 2:**
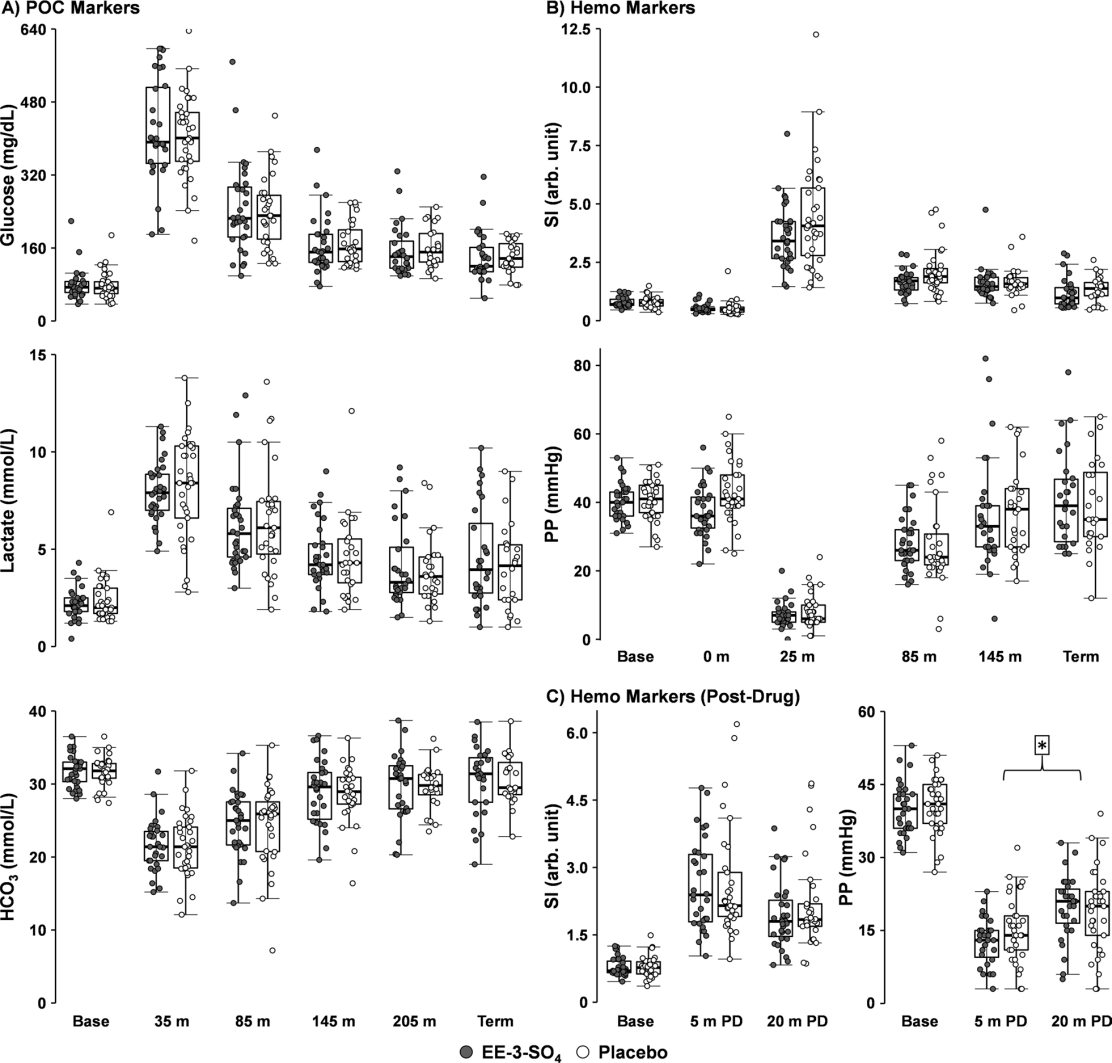
Box-and-scatter plots depicting primary point-of-care (POC; Panel **A**) and invasive hemodynamic (Hemo; Panels **B** and **C**) markers for EE-3-SO_4_ (filled circles) and Placebo (unfilled circles) cohorts. Data points are presented at collection times corresponding to Table 1, with POC measurements (glucose, lactate, bicarbonate [HCO_3_]) occurring at baseline (Base), following drug/Placebo administration (35 m), at hour intervals post-trauma (85 m, 145 m, 205 m), and at the terminal experimental endpoint (Term; ~ 295 min post-traumatic brain injury). Primary hemodynamic markers (shock-index [SI] and pulse-pressure [PP]) were continuously collected over the course of the entire experiment (Panel **B**), with data points displayed for baseline, immediately post-traumatic brain injury (0 m), immediately post blood loss procedure (25 m), at hour intervals post-trauma (85 m and 145 m), and at the terminal endpoint. Panel **C** presents a smaller temporal window to capture the predicted rapid effects (5 and 20 min post-drug) of EE-3-SO_4_ administration (significant Group × Time interaction). The asterisk denotes significant Group × Time interaction for PP

Results from primary point-of-care measurements of glucose, lactate and bicarbonate (Group × Time: see Additional file 1: Table S1 and Fig. 2A) were null after Bonferroni correction (0.05/3 tests; all *p*’s > 0.017, *d* =  − 0.12 to 0.16), as were secondary point-of-care variables (all *p*’s > 0.01, *d* =  − 0.14 to 0.23; Additional file 1: Fig. S2). Results indicated that there were no significant main effects or interactions (Group × Time: see Fig. 2B) between EE-3-SO_4_ and Placebo cohorts on SI or PP following Bonferroni correction (0.05/2 tests; all *p*’s > 0.025, *d* =  − 0.46 to − 0.15). Secondary variables of MAP and heart rate were null for all main effects and interactions as well (*d* =  − 0.33 to 0.17; Additional file 1: Fig. S3A). Please see Additional file 1 for expected main effects associated with Time.

A drug-focused analysis examining primary hemodynamic measures at 5 min and 20 min immediately post-drug administration (Fig. 2C) demonstrated a significant Group × Time interaction for PP (*F*_1,62.00_ = 6.33, *p* = 0.014). This interaction was driven by a more rapid recovery of PP in the EE-3-SO_4_ (*p* < 0.001; repeated measures *d* = 1.16) relative to the Placebo (*p* < 0.001; repeated measures *d* = 0.47) cohort. No Group effects or interactions were observed for SI values (0.05/2 tests; all *p*’s > 0.025, *d* =  − 0.13) or secondary hemodynamic markers of heart rate and MAP (0.05/2 tests; all *p*’s > 0.025, *d* =  − 0.07 to − 0.05; Additional file 1: Fig. S3B).

**Fig. 3:**
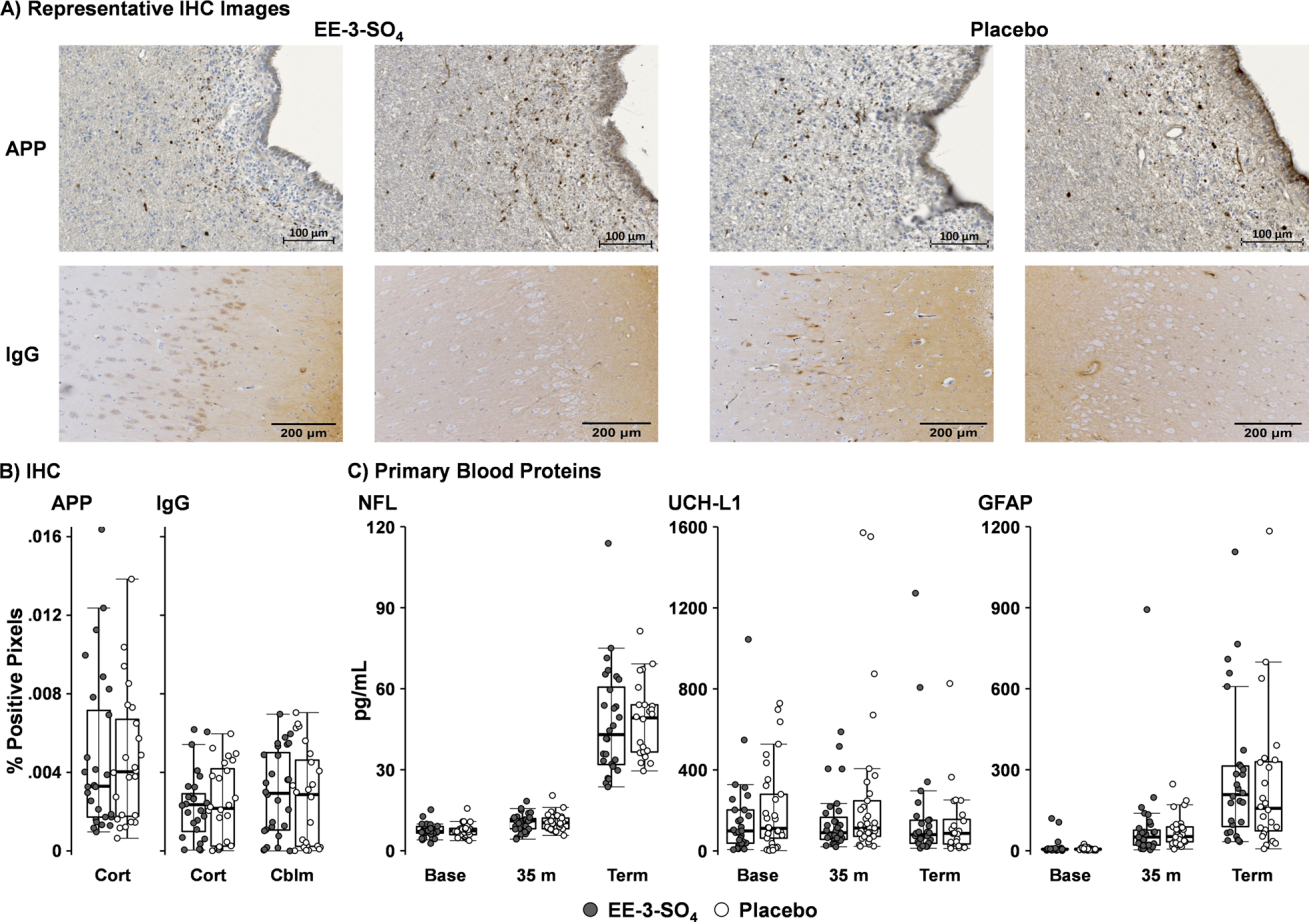
Panel **A** depicts immunohistochemistry (IHC) data for 2 animals from the EE-3-SO_4_ and Placebo cohorts of both amyloid precursor protein (APP) and Immunoglobulin G (IgG) at the level of the caudate nucleus near the second ventricle. Panel **B** depicts box-and-scatter plots of immunohistochemistry data for EE-3-SO_4_ (filled circles) and Placebo (unfilled circles) cohorts for the percentage of cortical (Cort) pixels presenting as positive for staining in both APP and IgG, including at the level of the cerebellum (Cblm) for IgG. Panel **C** presents box-and-scatter plots for primary blood biomarkers of glial fibrillary acidic protein (GFAP), neurofilament light chain (NFL), and ubiquitin carboxy-terminal hydrolase L1 (UCH-L1) collected at baseline (Base), following drug/Placebo administration (35 m), and at the terminal experimental endpoint (Term; ~ 295 min post-traumatic brain injury). None of the main effects or interactions associated with the Group variable (EE-3-SO_4_ vs. Placebo) were significant following Bonferroni correction

Immunohistochemical results (Fig. 3A, B) indicated no significant differences for EE-3-SO_4_ relative to Placebo for either axonal pathology (APP; cortex only) or BBB breach (IgG extravasation) in the cortex or cerebellum following Bonferroni correction (0.05/3 tests; all *p*’s > 0.017, *d* =  − 0.06 to 0.12). Independent 2 × 2 (Group × Time: 5 min post-injury vs. pre-terminal with skull sensor presence as a covariate) did not demonstrate any Group effects or interactions for primary markers (NFL/UCH-L1/GFAP; Fig. 3C) following Bonferroni correction (0.05/3 tests; all *p*’s > 0.017, *d* =  − 0.14 to 0.09). Secondary blood biomarkers (Aβ40 and Aβ42; Additional file 1: Fig. S4) indicated a Group × Time interaction for Aβ40 (*F*_1,53.80_ = 6.80, *p* = 0.012) that survived Bonferroni correction (0.05/2 = 0.025), whereas there was no Group effect or interaction for Aβ42 (*p*’s > 0.025; *d* =  − 0.25). The Aβ40 was characterized by a faster recovery in the EE-3-SO_4_ (*p* < 0.001; repeated measures *d* = 0.54) cohort across time relative to the Placebo (*p* < 0.001; repeated measures *d* = 0.26) cohort, partially driven by a higher Aβ40 value immediately post-drug for the EE-3-SO_4_ group.

### Secondary analyses

In terms of overall group composition, results indicated that there was approximately a twofold increase (*χ*^2^ = 8.17, *p* = 0.004) in the proportion of males in the non-surviving (13/16; 81.3%) versus the surviving group (21/52; 40.4%). There were no differences between the non-surviving and matched surviving samples for other demographics (age and weight) or indices of trauma (peak velocity, time to peak, deceleration time, time between TBI and end of blood loss, and blood loss volume). There were no differences between baseline point-of-care measures, invasive hemodynamic markers, or blood-based biomarkers (all *p*’s > 0.05).

**Fig. 4:**
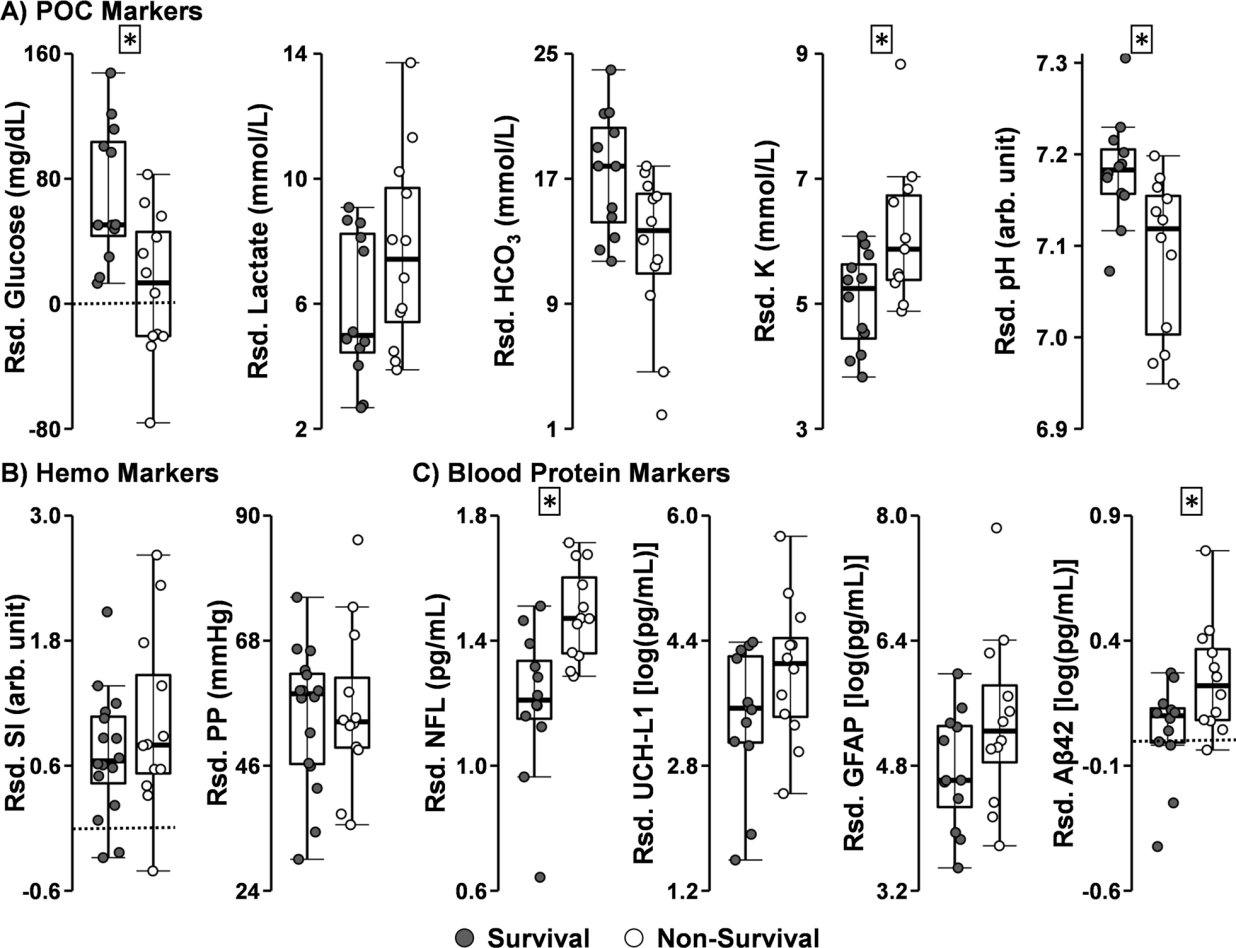
Box-and-scatter plots depicting all primary and significant secondary variables from point-of-care (POC; Panel **A**), invasive hemodynamic (Hemo; Panel **B**), and blood protein (Panel **C**) markers for survival analyses. All data were obtained from the last available, non-terminal timepoint for the non-surviving cohort, and the equivalent time point for each animal’s match (Surviving cohort). Graphed data have been residualized (Rsd.) to account for the effects of initial baseline values and varying measurement acquisition time post-injury. In the case of larger adjustments, this means that negative values are possible. Plots for primary POC measurements (glucose, lactate, bicarbonate [HCO_3_]), significant secondary POC measurements (potassium [K], potential hydrogen [pH]), primary hemodynamic measurements (shock-index [SI] and pulse-pressure [PP]), primary blood protein markers (neurofilament light chain [NFL], ubiquitin C-terminal hydrolase [UCH-L1], glial fibrillary acidic protein [GFAP]) and significant secondary protein markers (amyloid-beta 42 [Aβ42]) are displayed. Asterisks denote significant main effects of Survival Status

All point-of-care (Fig. 4A) and invasive hemodynamic measurement (Fig. 4B) analyses were performed on the last available measurement (prior to moribund criteria being met), and controlled for baseline levels and acquisition time post-injury. Glucose was the sole primary point-of-care variable exhibiting a significant effect, but was higher in surviving relative to non-surviving animals (*F*_1,20_ = 5.76, *p* = 0.026, *d* = 0.98), whereas HCO_3_ (*d* = 0.80) and lactate (*d* =  − 0.66) exhibited medium to large effect sizes but were non-significant at current sample sizes (both *p*’s > 0.05). Analysis of secondary point-of-care variables indicated significantly lower pH values (*F*_1,20_ = 7.26, *p* = 0.014, *d* = 1.13) and elevated K (*F*_1,19_ = 5.98, *p* = 0.024, *d* =  − 1.03) for non-surviving animals. No primary or secondary invasive hemodynamic measurement was significant for survival status (all *p*’s > 0.05, *d* =  − 0.40 to 0.66). NFL values (*F*_1,19_ = 12.72, *p* = 0.002, *d* =  − 1.46) were significantly higher for non-surviving animals among primary blood protein markers (Fig. 4C). Both GFAP (*d* =  − 0.72) and UCH-L1 (*d* =  − 0.74) were not statistically significant, but exhibited medium effect sizes in a similar direction indicating higher pathology (*p*’s > 0.05). Similarly, secondary blood-based biomarkers demonstrated significantly higher levels in non-surviving versus Surviving animals for Aβ42 (*F*_1,19_ = 5.66, *p* = 0.028, *d* =  − 0.97), with a null Group effect observed for Aβ40 (*p* > 0.05, *d* =  − 0.60).

## Discussion

Blood products are not always available in extreme circumstances [1, 36], necessitating the development of novel agents that can both augment the body’s natural response to severe blood loss and mitigate pathological aspects of shock [4]. The current study examined the efficacy of EE-3-SO_4_ as a treatment for TBI + HS in an austere environment (no mechanical ventilation post-injury, no additional resuscitation fluids, no craniotomy), as frequently occurs in military trauma scenarios and in developing countries [32]. The blocked randomization procedures adequately controlled for all potential major confounders from both demographic variables (non-significant differences in animal age, weight and sex) and experimental (statistically equivalent TBI exposure parameters, pre-injury anesthetic time, blood loss levels, etc.) procedures. Current results replicated previous findings of metabolic derangements, a decrease in MAP in conjunction with increased heart-rate, and both blood-based and immunohistochemical evidence of diffuse axonal injury and blood brain barrier disruption in a large animal model of closed-head accelerative TBI + HS [32].

The administration of EE-3-SO_4_ increased survival rate, normalized pulse pressure immediately post-drug, and provided preliminary evidence of neuroprotection relative to the Placebo cohort in this fully blinded trial. Previous studies have demonstrated increased survival rates and times for rodent and swine models of HS following intravenous EE-3-SO_4_ administration [15, 21]. Current findings extend these results to a large animal model of TBI + HS with approximately 40% blood loss, with EE-3-SO_4_ significantly prolonging survival rate relative to a control cohort (90.3% vs. 72.7%, respectively), albeit at a smaller magnitude relative to previous studies of isolated and severe HS [15]. The mortality rate observed in the Placebo cohort was also roughly commensurate with reported Class III–IV trauma rates [13], providing additional external validity for the closed-head TBI + HS model.

The majority of animals in both groups expired from respiratory distress rather than cardiovascular factors, similar to a previous TBI + HS swine model with 55% blood loss [32]. Acute respiratory failure represents the leading cause of death in preclinical models of isolated TBI [9], complicates clinical care of TBI patients [37], and is more common following TBI + HS relative to HS alone [10]. The current study did not directly quantify the presence of congestion, edema, hemorrhage or microatelectasis in pulmonary tissue as has been done in previous swine models [38], complicating the dissociation of central nervous system involvement in respiratory failure due to TBI. Mechanical ventilation remains the first line of defense for managing acute respiratory distress syndrome in both pre-hospital and hospital setting following complex trauma [39] and is typically used during all phases of preclinical trauma models [3]. However, mechanical ventilation is not available as a treatment option in austere environments to combat respiratory distress, representing a potentially critical factor that should be more carefully considered in future studies for full bench-to-bedside translation.

EE-3-SO_4_ also more rapidly restored pulse pressure post-administration relative to Placebo, followed by statistically equivalent pressures for the remainder of the experiment. The rapid action of EE-3-SO_4_ on pulse pressure suggests direct activation of estrogen receptors rather than through genomic signaling [20]. The membrane receptor effects of estrogen include activation of endothelial nitric oxide synthase and the consequent production of nitric oxide, as well as endothelial-independent, rapid mobilization and release of calcium within subcellular compartments leading to increases in Caþþ-triggered Kþ channel activity [22]. Activation of these receptors collectively results in changes to myocardial contractility and vasodilation of vascular smooth muscle [20]. Over-exuberant vasodilation in the face of severe hypovolemia could be detrimental, but initial vasodilation could also moderate the intense peripheral vasoconstriction seen in TBI + HS, and contribute to the normalization of pulse pressure.

Replication analyses indicated significant post-injury changes in all point-of-care markers of acidosis and other metabolic derangements (glucose, lactate, bicarbonate, etc.), the majority of which did not recover to baseline levels at the end of the 5 h monitoring period. Several of point-of-care markers (pH and potassium) were significantly more affected in non-surviving relative to surviving animals *prior to* death, although glucose was unexpectedly higher for surviving animals. Acidosis represents one of the hallmark complications of HS, and non-surviving animals were unable to compensate from a hemodynamic perspective, ultimately resulting in even further increases in oxygen debt at the tissue level [4, 40]. With the exception of glucose, current findings also partially replicate a previous swine model of severe hemorrhage, which reported that increased glucose/potassium/lactate and decreased bicarbonate/MAP were associated with survival [15]. In contrast to previous work in rodents [20], EE-3-SO_4_ administration did not significantly affect either point-of-care markers or MAP relative to Placebo, suggesting the need for polytherapeutic approaches to further promote survival and more rapidly restore homeostasis following TBI + HS [7].

Clinical research studies are increasingly using blood-based protein assays to characterize the extent of neurotrauma both in the acute and chronic injury phases of TBI [33]. Previous findings [32] of significant changes in NFL, GFAP and Aβ42 were replicated in our swine models of accelerative TBI + HS, with UCH-L1 and Aβ40 also significant in the current study due to increased statistical power. Several of these blood-based biomarkers demonstrated sensitivity to injury as soon as 35 min post-TBI and were strongly associated with survival, suggesting potential prognostic indications and a portable test for TBI [41]. Similarly, immunohistochemical evidence of diffuse axonal injury (periventricular region only) and blood–brain barrier breach (both periventricular and cerebellar regions) were also present, with previous research suggesting a close coupling between these pathologies [42–44]. In contrast, there were no significant differences between the Placebo and Sham cohorts on an immunohistochemical marker of inflammation (IBA1) following correction for multiple comparisons. The lack a neuroinflammatory response most likely reflects the relatively brief, 5-h post-injury monitoring period employed in the current study, as neuroinflammation has been shown to be present for multiple years post-injury following TBI [45].

Estrogen sulfate has been shown to increase cerebral perfusion pressure, increase partial brain oxygen pressure and decrease intracranial pressure, but not to affect markers of diffuse axonal injury in a previous rodent study [30]. Contrary to our a priori predictions, EE-3-SO_4_ showed evidence of normalizing plasma levels of Aβ40 rather than biomarkers traditionally associated with blood brain barrier breach or neuroinflammation. Aβ is a 40–42 amino acid long peptide generated by successive cleavage of amyloid pre-cursor protein by β-secretase followed by γ-secretase [44]. Although Aβ42 is believed to be more toxic, both forms have been shown to be rapidly released post-TBI, persist for weeks to months post-injury, and are typically viewed as potential markers of diffuse axonal injury [46, 47]. Numerous preclinical studies have suggested neuroprotective effects for 17β-estradiol [48], although estradiol is also elevated post-TBI and has been shown to confer an increased risk of death in severe human TBI [49]. However, it remains unknown whether the elevated levels of estradiol post-TBI are due to decreased metabolism (i.e., hydroxylation of estradiol to estrone or increased synthesis due to increased aromatase activity). Although promising, current findings of a more rapid recovery in plasma Aβ40 following EE-3-SO_4_ administration require further replication given the lack of efficacy for other female steroidal hormones in clinical TBI trials [50] and current null findings for APP immunohistochemistry.

In the current study, male sex was associated with a nearly twofold increase in mortality rate regardless of drug assignment. There is a rich preclinical literature suggesting that biological sex and associated female endogenous steroidal hormones affect systematic responses to both blunt force and neurotrauma, but with mixed findings in clinical studies [9, 40, 48, 51–53]. Specifically, retrospective clinical studies suggest that female sex may be protective against blunt-force trauma complications such as organ failure and sepsis rather than confer a benefit in terms of mortality [53–55]. Other clinical studies have suggested that only perimenopausal or postmenopausal females demonstrate decreased mortality following isolated moderate to severe TBI [56, 57], whereas pediatric-focused TBI studies indicated increased survival only for post-pubescent females [58, 59]. The latter more closely corresponds to the approximate age of the swine used in the current study and potentially suggests a U shaped relationship between female sex and neuroprotection as a function of age.

There are several limitations to the study that should be noted. First, the current study purposefully did not measure several physiological functions (cerebral perfusion pressure, partial brain oxygen pressure, intracranial pressure, etc.) due to their invasive nature. The study design was intentionally focused on point-of-care and blood based biomarkers that can readily be performed in humans relative to more sophisticated immunohistochemical assays, and our aim to examine a more realistic closed head injury (i.e., intact skull). Blood samples and embedded brain tissue from this study will be made available upon request for additional, secondary analyses. Second, all animals received anesthesia throughout the entire protocol in compliance with the approved ethical framework for this study. Although this is unlikely to have influenced drug-related outcomes due to the fully randomized and blinded design, it may have artificially inflated mortality rates associated with respiratory depression across both cohorts. The selected anesthetic regimen partially mitigated this confounder through utilization of agents that minimize respiratory depression (i.e., midazolam and ketamine) relative to isoflurane, but in doing so also potentially increased neuroprotective effects [60]. Finally, animals were only monitored for up 5 h post-injury in the current study, which limits the conclusions that can be drawn about more long-term therapeutic effects of EE-3-SO_4_ or long-term pathophysiological consequences of the TBI + HS model.

## Conclusions

In summary, blood products (whole blood, plasma, etc.) represent the treatment of choice for severe blood loss with or without a concomitant TBI, but are not always available [11, 12]. Current results provide additional support for the efficacy of EE-3-SO_4_ to promote survival following HS and TBI + HS in austere environments in the absence of fluid resuscitation [15, 20, 21], along with additional salutary effects on hemodynamics. Poly-therapeutic approaches that target additional mechanisms (increased hemostasis, oxygen carrying capacity, etc.) for promoting survival to complement the beneficial effects of EE-3-SO_4_ should be considered in future studies, along with more in-depth characterization of how EE-3-SO_4_ potentially mitigates neuronal and pulmonary injury.

## Supplementary Information

**Additional file 1.** Supplemental Materials.

## Data Availability

The data sets used and/or analyzed during the current study are available from the corresponding author on request.

## Abbreviations

Aβ40: Amyloid beta 40
Aβ42: Amyloid beta 42
APP: Amyloid precursor protein
BBB: Blood–brain barrier
EE-3-SO_4_: Estradiol-3-sulfate
GFAP: Glial fibrillary acidic protein
GLM: General linear models
HCO_3_: Bicarbonate
HS: Hemorrhagic shock
IBA1: Ionized calcium-binding adaptor molecule 1
iCa: Ionized calcium
IgG: Immunoglobulin G
K: Potassium
LME: Linear mixed effects
MAP: Mean arterial pressure
Na: Sodium
NFL: Neurofilament light
PCO_2_: Partial CO_2_ pressure
pH: Potential hydrogen
PP: Pulse pressure
SI: Shock index
TBI: Traumatic brain injury
UCH-L1: Ubiquitin C-terminal hydrolase L1

## References

[1] Eastridge BJ, Mabry RL, Seguin P, Cantrell J, Tops T, Uribe P (2012). Death on the battlefield (2001–2011): implications for the future of combat casualty care. J Trauma Acute Care Surg.

[2] Shafi S, Collinsworth AW, Richter KM, Alam HB, Becker LB, Bullock MR (2016). Bundles of care for resuscitation from hemorrhagic shock and severe brain injury in trauma patients—translating knowledge into practice. J Trauma Acute Care Surg.

[3] Mayer AR, Dodd AB, Vermillion MS, Stephenson DD, Chaudry IH, Bragin DE (2019). A systematic review of large animal models of combined traumatic brain injury and hemorrhagic shock. Neurosci Biobehav Rev.

[4] Cannon JW (2018). Hemorrhagic shock. N Engl J Med.

[5] Matsushita Y, Bramlett HM, Kuluz JW, Alonso O, Dietrich WD (2001). Delayed hemorrhagic hypotension exacerbates the hemodynamic and histopathologic consequences of traumatic brain injury in rats. J Cereb Blood Flow Metab.

[6] Vella MA, Crandall ML, Patel MB (2017). Acute management of traumatic brain injury. Surg Clin North Am.

[7] Muller CR, Courelli V, Lucas A, Williams AT, Li JB, Dos SF (2021). Resuscitation from hemorrhagic shock after traumatic brain injury with polymerized hemoglobin. Sci Rep.

[8] Gutierrez G, Das A, Ballarino G, Beyzaei-Arani A, Turkan H, Wulf-Gutierrez M (2013). Decreased respiratory rate variability during mechanical ventilation is associated with increased mortality. Intensive Care Med.

[9] Xiong Y, Mahmood A, Chopp M (2013). Animal models of traumatic brain injury. Nat Rev Neurosci.

[10] Galvagno SM, Fox EE, Appana SN, Baraniuk S, Bosarge PL, Bulger EM (2017). Outcomes after concomitant traumatic brain injury and hemorrhagic shock: a secondary analysis from the pragmatic, randomized optimal platelets and plasma ratios trial. J Trauma Acute Care Surg.

[11] Butler FK, Holcomb JB, Shackelford S, Barbabella S, Bailey JA, Baker JB (2018). Advanced resuscitative care in tactical combat casualty care: TCCC guidelines change 18–01:14 October 2018. J Spec Oper Med.

[12] Callcut RA, Kornblith LZ, Conroy AS, Robles AJ, Meizoso JP, Namias N (2019). The why and how our trauma patients die: a prospective Multicenter Western Trauma Association study. J Trauma Acute Care Surg.

[13] American College of Surgeons Committee on Trauma (2004). Advanced trauma life support program for doctors.

[14] Yu HP, Chaudry IH (2009). The role of estrogen and receptor agonists in maintaining organ function after trauma-hemorrhage. Shock.

[15] Miller M, Keith J, Berman J, Burlington DB, Grudzinskas C, Hubbard W (2014). Efficacy of 17alpha-ethynylestradiol-3-sulfate for severe hemorrhage in minipigs in the absence of fluid resuscitation. J Trauma Acute Care Surg.

[16] Ardelt AA, McCullough LD, Korach KS, Wang MM, Munzenmaier DH, Hurn PD (2005). Estradiol regulates angiopoietin-1 mRNA expression through estrogen receptor-alpha in a rodent experimental stroke model. Stroke.

[17] Hubbard WJ, Chaudry IH, Heidenreich K (2017). The use of estrogen for the treatment of traumatic brain injury. New therapeutics for traumatic brain injury.

[18] Chen JG, Not L, Ng T, Hubbard WJ, Chatham J, Choudhry MA (2006). 17beta-estradiol(E2) administration after major blood loss improves liver ATP, 3-h survival and also long-term survival following prolonged hypotension (3 h) and fluid resuscitation. Shock.

[19] Kim H, Chen J, Zinn KR, Hubbard WJ, Fineberg NS, Chaudry IH (2010). Single photon emission computed tomography demonstrated efficacy of 17ß-estradiol therapy in male rats after trauma-hemorrhage and extended hypotension. J Trauma Acute Care Surg.

[20] Hubbard WJ, Yang S, Chaudry IH (2021). Ethinyl estradiol sulfate acts without fluid resuscitation through estrogen receptors to rapidly protect the cardiovascular system from severe hemorrhage. J Trauma Acute Care Surg.

[21] Hubbard W, Keith J, Berman J, Miller M, Scott C, Peck C (2015). 17alpha-Ethynylestradiol-3-sulfate treatment of severe blood loss in rats. J Surg Res.

[22] Yu X, Ma H, Barman SA, Liu AT, Sellers M, Stallone JN (2011). Activation of G protein-coupled estrogen receptor induces endothelium-independent relaxation of coronary artery smooth muscle. Am J Physiol Endocrinol Metab.

[23] Hsieh YC, Yu HP, Suzuki T, Choudhry MA, Schwacha MG, Bland KI (2006). Upregulation of mitochondrial respiratory complex IV by estrogen receptor-beta is critical for inhibiting mitochondrial apoptotic signaling and restoring cardiac functions following trauma-hemorrhage. J Mol Cell Cardiol.

[24] Lee SJ, McEwen BS (2001). Neurotrophic and neuroprotective actions of estrogens and their therapeutic implications. Annu Rev Pharmacol Toxicol.

[25] Sohrabji F (2007). Guarding the blood–brain barrier: a role for estrogen in the etiology of neurodegenerative disease. Gene Expr.

[26] Ardelt AA, Anjum N, Rajneesh KF, Kulesza P, Koehler RC (2007). Estradiol augments peri-infarct cerebral vascular density in experimental stroke. Exp Neurol.

[27] O'Donnell ME, Lam TI, Tran LQ, Foroutan S, Anderson SE (2006). Estradiol reduces activity of the blood–brain barrier Na–K–Cl cotransporter and decreases edema formation in permanent middle cerebral artery occlusion. J Cereb Blood Flow Metab.

[28] Akabori H, Moeinpour F, Bland KI, Chaudry IH (2010). Mechanism of the anti-inflammatory effect of 17beta-estradiol on brain following trauma-hemorrhage. Shock.

[29] Day NL, Floyd CL, D'Alessandro TL, Hubbard WJ, Chaudry IH (2013). 17beta-estradiol confers protection after traumatic brain injury in the rat and involves activation of G protein-coupled estrogen receptor 1. J Neurotrauma.

[30] Kim H, Cam-Etoz B, Zhai G, Hubbard WJ, Zinn KR, Chaudry IH (2015). Salutary effects of estrogen sulfate for traumatic brain injury. J Neurotrauma.

[31] Taylor CA, Bell JM, Breiding MJ, Xu L (2017). Traumatic brain injury-related emergency department visits, hospitalizations, and deaths—United States, 2007 and 2013. MMWR Surveill Summ.

[32] Mayer AR, Dodd AB, Ling JM, Stephenson DD, Rannou-Latella JG, Vermillion MS (2021). Survival rates and biomarkers in a large animal model of traumatic brain injury combined with two different levels of blood loss. Shock.

[33] Zetterberg H, Blennow K (2016). Fluid biomarkers for mild traumatic brain injury and related conditions. Nat Rev Neurol.

[34] du Percie SN, Hurst V, Ahluwalia A, Alam S, Avey MT, Baker M, et al. The ARRIVE guidelines 2.0: updated guidelines for reporting animal research. Br J Pharmacol. 2020;177(16):3617–24. 10.1111/bph.15193 PMC7393194 32662519

[35] Mayer AR, Ling JM, Dodd AB, Rannou-Latella JG, Stephenson DD, Dodd RJ (2021). Reproducibility and characterization of head kinematics during a large animal acceleration model of traumatic brain injury. Front Neurol.

[36] Koh EY, Oyeniyi BT, Fox EE, Scerbo M, Tomasek JS, Wade CE (2019). Trends in potentially preventable trauma deaths between 2005–2006 and 2012–2013. Am J Surg.

[37] Hendrickson CM, Howard BM, Kornblith LZ, Conroy AS, Nelson MF, Zhuo H (2016). The acute respiratory distress syndrome following isolated severe traumatic brain injury. J Trauma Acute Care Surg.

[38] Scultetus AH, Jefferson MA, Haque A, Hubbell JN, Arnaud FG, Moon-Massat P (2020). Histopathological evidence of multiple organ damage after simulated aeromedical evacuation in a wwine acute lung injury model. Mil Med.

[39] Noorbakhsh MR, Kriley IR (2018). Management of severe respiratory failure in complex trauma patients. J Emerg Crit Care Med March.

[40] Hildebrand F, Andruszkow H, Huber-Lang M, Pape HC, van Griensven M (2013). Combined hemorrhage/trauma models in pigs-current state and future perspectives. Shock.

[41] Wang KK, Yang Z, Zhu T, Shi Y, Rubenstein R, Tyndall JA (2018). An update on diagnostic and prognostic biomarkers for traumatic brain injury. Expert Rev Mol Diagn.

[42] Johnson VE, Weber MT, Xiao R, Cullen DK, Meaney DF, Stewart W (2018). Mechanical disruption of the blood–brain barrier following experimental concussion. Acta Neuropathol.

[43] Wojnarowicz MW, Fisher AM, Minaeva O, Goldstein LE (2017). Considerations for experimental animal models of concussion, traumatic brain injury, and chronic traumatic encephalopathy—these matters matter. Front Neurol.

[44] Karran E, De SB (2016). The amyloid cascade hypothesis: are we poised for success or failure?. J Neurochem.

[45] Johnson VE, Stewart JE, Begbie FD, Trojanowski JQ, Smith DH, Stewart W (2013). Inflammation and white matter degeneration persist for years after a single traumatic brain injury. Brain.

[46] Johnson VE, Stewart W, Smith DH (2012). Widespread tau and amyloid-beta pathology many years after a single traumatic brain injury in humans. Brain Pathol.

[47] Washington PM, Morffy N, Parsadanian M, Zapple DN, Burns MP (2014). Experimental traumatic brain injury induces rapid aggregation and oligomerization of amyloid-beta in an Alzheimer's disease mouse model. J Neurotrauma.

[48] Kovesdi E, Szabo-Meleg E, Abraham IM (2020). The role of estradiol in traumatic brain injury: mechanism and treatment potential. Int J Mol Sci.

[49] Kumar RG, DiSanto D, Awan N, Vaughan LE, Levochkina MS, Weppner JL (2020). Temporal acute serum estradiol and tumor necrosis factor-alpha associations and risk of death after severe traumatic brain injury. J Neurotrauma.

[50] Wright DW, Yeatts SD, Silbergleit R, Palesch YY, Hertzberg VS, Frankel M (2014). Very early administration of progesterone for acute traumatic brain injury. N Engl J Med.

[51] Mizushima Y, Wang P, Jarrar D, Cioffi WG, Bland KI, Chaudry IH (2000). Estradiol administration after trauma-hemorrhage improves cardiovascular and hepatocellular functions in male animals. Ann Surg.

[52] Semenas E, Nozari A, Sharma HS, Basu S, Rubertsson S, Wiklund L (2010). Sex differences in cerebral injury after severe haemorrhage and ventricular fibrillation in pigs. Acta Anaesthesiol Scand.

[53] Trentzsch H, Nienaber U, Behnke M, Lefering R, Piltz S (2014). Female sex protects from organ failure and sepsis after major trauma haemorrhage. Injury.

[54] George RL, McGwin G, Windham ST, Melton SM, Metzger J, Chaudry IH (2003). Age-related gender differential in outcome after blunt or penetrating trauma. Shock.

[55] Sperry JL, Nathens AB, Frankel HL, Vanek SL, Moore EE, Maier RV (2008). Characterization of the gender dimorphism after injury and hemorrhagic shock: are hormonal differences responsible?. Crit Care Med.

[56] Berry C, Ley EJ, Tillou A, Cryer G, Margulies DR, Salim A (2009). The effect of gender on patients with moderate to severe head injuries. J Trauma.

[57] Hong ZJ, Firek M, Zachary B, Mors K, Schindler C, Marzi I, et al. The effect of age and sex on outcomes following isolated moderate to severe traumatic brain injury. Eur J Trauma Emerg Surg. 2020. 10.1007/s00068-020-01491-1 32929551

[58] Ley EJ, Short SS, Liou DZ, Singer MB, Mirocha J, Melo N (2013). Gender impacts mortality after traumatic brain injury in teenagers. J Trauma Acute Care Surg.

[59] Phelan HA, Shafi S, Parks J, Maxson RT, Ahmad N, Murphy JT (2007). Use of a pediatric cohort to examine gender and sex hormone influences on outcome after trauma. J Trauma.

[60] Chang LC, Raty SR, Ortiz J, Bailard NS, Mathew SJ (2013). The emerging use of ketamine for anesthesia and sedation in traumatic brain injuries. CNS Neurosci Ther.

