# Supplementary material for: 17α-Ethinyl estradiol-3-sulfate increases survival and hemodynamic functioning in a large animal model of combined traumatic brain injury and hemorrhagic shock: a randomized control trial
Source: Crit Care. 2021 Dec 16;25:428. doi: 10.1186/s13054-021-03844-7 (PMC8675515; doi:10.1186/s13054-021-03844-7)
Supplement: Supplementary file 1 — Additional file 1. Supplemental Materials. [file 13054_2021_3844_MOESM1_ESM.docx]

**Supplemental Materials for ‘17α-Ethinyl Estradiol-3-Sulfate Increases Survival and Hemodynamic Functioning in a Large Animal Model of Combined Traumatic Brain Injury and Hemorrhagic Shock: A Randomized Control Trial’**

**Methods**

*Animal Preparation*

All in-life study procedures occurred between April 2019 and February 2020 in 5 separate animal cohorts. Animals were initially sedated with midazolam (0.5 mg/kg IM injection), pre-medicated with buprenorphine (0.02 mg/kg IM), and maintained under general anesthesia using a combination of isoflurane, (5% induction, 1-4% for maintenance combined with oxygen; during surgical procedures and as needed post-TBI), midazolam (0.1-0.5 mg/kg IV bolus immediately pre-TBI; 0.5 mg/kg/hour to 1.5 mg/kg/hour IV as needed post-TBI) and ketamine (8 mg/kg/hour to 15 mg/kg/hour IV as needed starting at 2 hours post- EE-3-SO_4_ or placebo treatment). Both femoral arteries were catheterized for invasive blood pressure monitoring, controlled blood loss, and arterial blood sampling. Catheters were flushed every 20 minutes in conjunction with a single artificial breath. In order to mimic an austere care environment with minimal treatment resources, no other additional care was administered.

Blood samples were obtained and analyzed using point-of-care devices (i-STAT, CG8+ cartridges; Abbott Point of Care Inc., Princeton, NJ; Lactate Plus, Nova Biomedical Corp., Waltham, MA) for primary (glucose, lactate and bicarbonate [HCO_3_]) and secondary (potential hydrogen [pH], partial CO_2_ pressure [PCO_2_], sodium [Na], potassium [K], ionized calcium [iCa]) variables. Plasma samples were centrifuged for 10 minutes at 2500 relative centrifugal force, aliquoted into 400-500 µl tubes, and immediately stored in an -80°C freezer. Protein concentrations in plasma samples were measured in duplicate with a Simoa HD-1 Analyzer (Quanterix Simoa, Billerica, MA) for neurofilament light (NFL), ubiquitin C-terminal hydrolase L1 (UCH-L1), and glial fibrillary acidic protein (GFAP; primary outcome measures), and for amyloid beta 40 and 42 (Aβ40 and Aβ42; secondary outcome measures). Non-baseline samples with a coefficient of variation (CV) higher than 30% were excluded from subsequent analyses.

Invasive arterial pressure monitoring (IOX v2.9.5.73, emka Technologies, Paris, France) was used to capture heart rate (HR), systolic and diastolic blood pressure, which was subsequently used to calculate primary (shock index [SI]; pulse pressure [PP]) and secondary (mean arterial pressure [MAP] and HR) hemodynamic outcome variables. Hemodynamic data were smoothed and averaged over 30-second epochs. The SI was calculated by dividing HR by systolic blood pressure, whereas PP was calculated by subtracting diastolic from systolic blood pressure. All epochs were physically examined to determine an appropriate beat threshold and verify blood pressure waveform fit by IOX. Average primary and secondary outcome variables are reported for the last 30 seconds of the baseline epoch (approximately 15 minutes prior to TBI), immediately following reestablishment of monitoring post-TBI, during the second phase of blood loss, and at approximately one-hour intervals up to three hours after termination of the blood loss procedure to maximally correspond with blood draws. Monitoring was not feasible while preparing animals for transport or during transport due to motion artifact. Focused analyses examining the potential effects of EE-3-SO_4_ on invasive hemodynamic measures (primary mechanism of action) were also performed 5 minutes and 20 minutes immediately post-administration of drug or placebo.

A closed-head TBI was initiated via a pneumatic device (HYGE, Inc., Kittanning, PA, USA) targeting a rotation in the coronal plane at a peak angular velocity (primary outcome) of 250 radians/second. Angular velocity for the TBI was acquired via an in-house data acquisition system using an ARS-06 angular rate sensor rigidly mounted to the arm of the device (Applied Technologies Associates, Albuquerque, NM, USA) at a 25 kHz sampling frequency (1). A subset of animals (N=19 EE-3-SO_4_; N=18 Placebo) had a lightweight sensor/mounting plate directly affixed to the skull (14 mm cortical screws) to more accurately capture head kinematics (2). All animals were placed in the sphynx position and secured to the HYGE device with a custom-made bite bar that was used in conjunction with two padded metal straps to convert linear to an angular motion (2). Time-to-peak (start time defined as 5% of peak velocity) and deceleration time (peak time to return to 5% of peak velocity) were identified as secondary variables. Sensor data were smoothed with a 4-pole, Butterworth filter (channel frequency class 1000) prior to calculations (3).

Immediately following the TBI exposure, animals were placed in lateral recumbency and subjected to arterial hemorrhage via controlled removal of approximately 40% of estimated total blood volume (eTBV=weight × 65 mL/kg) via a peristaltic pump (Harvard Apparatus, Holliston, MA, USA). Blood loss occurred 161±48.7 seconds after the TBI (range 88-330 seconds) in a biphasic fashion (20% eTBV in 7.5 minutes; 20% in 15 minutes) to better approximate the normal physiological response during severe blood loss. Total blood loss volume was subsequently confirmed by total blood weight (scalar factor=1.06 g/mL). Animals were then administered EE-3-SO_4_ (formulated in the vehicle at 5.0 mg/mL and dosed at 0.2 mL/kg) or placebo (0.45% sodium chloride solution) as an intravenous administration over 5 minutes via a continuous pump (0.2 mL/kg). Animals were monitored up to approximately 5 hours post-TBI.

*Tissue Handling and Neuropathological Evaluation*

Single immunohistochemistry labeling was performed to examine for extravasated serum proteins (Immunoglobulin G; IgG) as markers of blood-brain barrier integrity, microglial activation (ionized calcium-binding adaptor molecule; IBA1) and axonal pathology (amyloid precursor protein; APP). All immunohistopathological analyses were performed on 8-micron thick sections, focusing at the level of the head of the caudate nucleus (APP, IgG, and IBA1) and at the mid vermis of the cerebellum (IgG) based on previous findings (1). All sections were visualized with 3, 3’-diaminobenzidine (DAB), counterstained with hematoxylin, dehydrated, and coverslipped. A no primary control and a positive tissue control were included in each experiment. Immunostained sections were imaged using an Olympus IX71 microscope.

A semi-automated process for APP N-terminus label quantification was carried out using macro code to allow for simultaneous batch processing of all images. The Color Deconvolution plugin in ImageJ Fiji (NIH, Bethesda, MD) was first used to separate the hematoxylin stain from the DAB stain by converting the original RGB image into three 8-bit images based on the vector colors for each stain. The resulting DAB image was then converted to grayscale and color-inverted. A threshold, determined by calculating the intensity of the lightest APP-positive axonal bulb, was applied to the image to remove background staining. The ImageJ Fiji cell counter plugin was utilized to track the count per unit area (877×660 µm). For IgG quantification, six images were taken adjacent to the sulcal depths at the level of the caudate nucleus in addition to three cerebellar images. IgG extravasation was quantified by performing color deconvolution to separate the DAB stain from the hematoxylin stain, calculating percentage of pixels over a consistent pre-determined background threshold, and then averaging the images. Six images were also taken in the radiation of the corpus callosum at the level of the caudate nucleus for IBA1 and quantified using methods identical to those described for IgG.

*Statistical Plan*

Generalized linear models (GLM) or linear mixed effects (LME) models were utilized for analyses with appropriate (Gaussian or Gamma) response distributions determined by the model fit. Cohorts were compared on key animal characteristics and baseline physiology to ensure effective matching using two-sided tests (4). All hemodynamic and point-of-care blood data were compared with LME using an unstructured covariance matrix to account for covariate dependent (Group) missingness (i.e., differences in survival rates) and to control for baselines values. Neural biomarker data were log-transformed following the addition of a constant prior to analyses due to large differences in variance across metrics. Neuronal biomarker analyses included the absence/presence of a head plate for skull sensor data collection.

The following quality assurance decisions were made prior to unblinding or data analyses. First, all terminal samples from non-surviving animals were excluded from analyses due to extreme physiological derangement (e.g., 5 minutes of apnea) and non-standard data collection times. Second, any animal that did not survive at least 20 minutes post-blood loss was excluded from subsequent drug-focused analyses. Third, all point-of-care (POC) data with machine-determined, out-of-range values were excluded. Fourth, any data that were collected with minor variations in protocol (e.g., sample collected 10 minutes past specified timepoint or recollected due to diluted sample) were individually reviewed for outlier status (none observed).

Analyses comparing EE-3-SO_4_ relative to Placebo cohorts were repeated for any variables that contained extreme outliers (3 × interquartile range) with the outliers removed. However, results were not significantly affected by the presence/absence of outliers for any of the repeated tests. Baseline tests were not corrected for multiple comparisons to maintain a more liberal threshold during examination of potential failures in randomization. All other omnibus analyses were Bonferroni-corrected based on the number of comparisons performed within each data type with the exception of the exploratory survival analyses.

Different quality assurance metrics were also performed for each individual data type. Invasive hemodynamic data from one animal (placebo) exhibited poor signal-to-noise, was identified as an extreme outlier and excluded. Two brain tissue samples with severe tears or artifacts preventing accurate estimates were excluded (one drug and one placebo animal), while those tissue samples demonstrating minor tears and/or unusual cellular morphology were examined for outlier status (none observed).

**Results**

***Characterization of Model Pathology***

Significant Group × Time interactions were observed for secondary point-of-care variables including Na (F_4,32.52_=13.50, *p*<0.001) and K (F_4,29.52_=8.63, *p*<0.001). Follow up tests indicated that Na was initially decreased in the Placebo group following blood loss (all *p*’s<0.001; Cohen’s *d* =-2.62 to -1.37), but demonstrated evidence of recovery from a statistical perspective (i.e., no group differences; *p*=0.364). The temporal trajectory for K was more complex, and indicated an initial increase in Placebo relative to Sham animals at 35 minutes post-TBI (*p*<0.001, *d*=2.56), no group differences at 85 minutes post-TBI, and then increased values from 145 minutes onward (all *p*’s < 0.049, *d*=0.79 to 1.25). Main effects of Group were observed for pH (F_1,26.39_=12.12, *p*=0.002, *d*=-1.16), PCO_2_ (F_1,25.48_=9.97, *p*=0.004, *d*=-1.04) and iCa (F_1,38.26_=41.82, *p*<0.001, *d*=2.15), with decreased values in Placebo relative to Sham animals. Main effects of Time for these variables (all *p*’s ≤ 0.008) were characterized by a gradual and complete return to typical values over time (PCO_2_), a large decrease in values up to 85 minutes post-TBI followed by a gradual but incomplete recovery (iCa), or a more variable recovery trajectory (pH).

Group × Time interactions were observed for secondary invasive hemodynamic measurements of HR (F_4,35.49_=18.86, *p*<0.001) and MAP (F_4,31.83_=59.79, *p*<0.001). Follow up tests indicated that HR was statistically equivalent between Placebo and Sham cohorts immediately post-TBI (*p*=0.364), but was then elevated in Placebo animals up to 295 minutes (all *p*’s *<* 0.001, *d*=1.81 to 2.50) with evidence of incomplete recovery. Similar to SI, MAP was elevated immediately post-TBI in the Placebo cohort (*p*=0.002, *d*=1.14), dramatically declined following blood loss (*p*<0.001, *d*=-5.43), and exhibited evidence of an incomplete recovery relative to the Sham cohort for the remaining timepoints (all *p*’s≤0.002, *d* =-1.76 to -1.21).

Secondary blood-based biomarker Aβ42 exhibited a significant Group × Time interaction (F_1,25.29_=7.53, *p*=0.011), characterized by significantly increased levels in Placebo relative to Shams at 35 minutes post-TBI (*p*<0.001; *d*= 2.09) as well as at 295 minutes post-TBI, albeit at a lesser effect (*p*=0.003; *d*= 1.19). The main effect of group was significant for Aβ40 (F_1,32.22_=20.23, *p*<0.001; *d*= 1.51) with increased values observed in the Placebo cohort.

*Main Effects of Time during EE-3-SO_4_ Analyses*

For point-of-care measures, main effects of Time were present for all primary (HCO_3_ [F_4,46.38_=67.23, *p*≤0.001], lactate [F_4,46.50_=48.18, p≤0.001], glucose [F_4,56.18_=154.72, *p*≤0.001]) and most secondary blood-based biomarkers (PCO_2_ [F_4,48.79_=21.98, p≤0.001], iCa [F_4,54.97_=30.44,p≤0.001], pH [F_4,48.23_=9.05,p≤0.001], Na [F_4,54.47_=110.99, *p*≤0.001], and K [F_4,42.73_=36.05, *p*≤0.001]). Similar to the patterns observed during characterization of model pathology, results generally fell into patterns of decreased values post-trauma without evidence of recovery to baseline (HCO_3_ and iCa), increased values without evidence of recovery (lactate and glucose), decreased values with evidence of recovery (Na and PCO_2_) or more variable patterns (pH and K). For the latter, both pH (decreased) and K (increased) showed evidence of pathology immediately post-trauma followed by partial recovery towards baseline values, only to deteriorate again towards the end of the experiment.

For hemodynamic measures, main effects of Time were present for primary (SI: F_4,46.49_=76.11, *p*<0.001; PP: F_4,53.25_=309.91, *p*<0.001) and secondary (HR: F_4,56.72_=111.36, *p*<0.001; MAP: F_4,45.63_=588.43, *p*<0.001) markers. Similar to the patterns observed during characterization of model pathology, SI and HR both increased following the blood loss procedure and showed evidence of an incomplete recovery to baseline levels. In contrast, PP decreased following blood loss and recovered to approximate baseline levels whereas MAP showed a more complex trajectory (increase post-TBI, decrease post blood loss, and near baseline recovery).

NFL (F_1,53.05_=763.88, *p*<0.001; *d*=-4.10) and GFAP (F_1,48.82_=105.77, *p*<0.001; *d*=-1.25) exhibited a main effect of Time associated with increasing values as a function of time. In contrast, Aβ40 (F_1,53.80_=91.01, *p*<0.001) and Aβ42 (F_1,52.90_=121.36, *p*<0.001) decreased with Time. The Time effect for UCH-L1 did not meet conventional levels of statistical significance (*p*=0.068).

**References**

(1) Mayer AR, Dodd AB, Ling JM, Stephenson DD, Rannou-Latella JG, Vermillion MS, et al. Survival rates and biomarkers in a large animal model of traumatic brain injury combined with two different levels of blood loss. Shock 2021;55(4):554-62.

(2) Mayer AR, Ling JM, Dodd AB, Rannou-Latella JG, Stephenson DD, Dodd RJ, et al. Reproducibility and Characterization of Head Kinematics During a Large Animal Acceleration Model of Traumatic Brain Injury. Frontiers in neurology 2021, 12:658461.

(3) Society of Automotive Engineers. SAE J211-1 (1995): Instrumentation for Impact Test, Part 1, Electronic Instrumentation. Society of Automotive Engineers, Inc; 1995.

(4) Smith DH, Hicks RR, Johnson VE, Bergstrom DA, Cummings DM, Noble LJ, et al. Pre-clinical traumatic brain injury common data elements: Toward a common language across laboratories. J Neurotrauma 2015;32(22):1725-35.

| Supplemental Table 1: Point-of-care blood measurements. | | | | | | | | | | | | | | | | | | | | | | | | | | |
| --- | --- | --- | --- | --- | --- | --- | --- | --- | --- | --- | --- | --- | --- | --- | --- | --- | --- | --- | --- | --- | --- | --- | --- | --- | --- | --- |
|  | Baseline | | | ~35 min | | | | ~85 min | | | | ~145 min | | | | ~205 min | | | | Terminal | | | |  | |  |
| i-STAT | Placebo (N=33) | EE-3-SO_4_ (N=31) | | Placebo (N=33) | | EE-3-SO_4_ (N=31) | | Placebo (N=31) | | EE-3-SO_4_ (N=31) | | Placebo (N=27) | | EE-3-SO_4_ (N=29) | | Placebo (N=24) | | EE-3-SO_4_ (N=28) | | Placebo (N=23) | | EE-3-SO_4_ (N=27) | | Interaction  *p* | |  |
| Primary Measures | | |  | |  | |  | |  | |  | |  | |  | |  | |  | |  | |  | |  | |
| Glucose (mg/dL) | 77.42±29.94 | 80.35±33.65 | | 404.76±92.68 | | 417.48±112.24 | | 241.06±77.70 | | 245.77±99.99 | | 169.93±46.22 | | 166.87±64.42 | | 159.46±44.05 | | 154.57±55.09 | | 139.87±34.93 | | 140.50±55.05 | | 0.971 | |  |
| Lactate (mmol/L) | 2.36±1.13 | 2.17±0.79 | | 8.25±2.67 | | 7.98±1.55 | | 6.45±2.75 | | 6.22±2.29 | | 4.58±2.08 | | 4.65±1.74 | | 3.89±1.76 | | 4.15±2.12 | | 4.12±2.25 | | 4.60±2.57 | | 0.979 | |  |
| HCO_3_ (mmol/L) | 31.69±2.06 | 31.78±2.18 | | 21.45±4.26 | | 21.61±3.60 | | 24.44±5.61 | | 24.64±4.36 | | 28.64±4.00 | | 28.88±4.38 | | 29.63±3.06 | | 29.74±4.52 | | 30.51±3.39 | | 30.46±4.72 | | 0.723 | |  |
| Secondary Measures | | |  | |  | |  | |  | |  | |  | |  | |  | |  | |  | |  | |  | |
| PCO_2_ (mm Hg) | 52.73±5.82 | 55.08±7.53 | | 39.44±13.70 | | 36.96±6.92 | | 43.28±6.84 | | 42.10±5.56 | | 46.45±8.31 | | 50.31±9.44 | | 46.78±8.25 | | 50.00±9.88 | | 57.58±12.07 | | 56.40±13.44 | | 0.095 | |  |
| iCa (mmol/L) | 1.38±0.05 | 1.41±0.05 | | 1.28±0.07 | | 1.27±0.05 | | 1.23±0.08 | | 1.23±0.06 | | 1.29±0.08 | | 1.30±0.06 | | 1.31±0.06 | | 1.32±0.06 | | 1.32±0.05 | | 1.33±0.05 | | 0.546 | |  |
| pH | 7.39±0.05 | 7.37±0.05 | | 7.35±0.11 | | 7.38±0.08 | | 7.35±0.09 | | 7.37±0.06 | | 7.40±0.07 | | 7.37±0.08 | | 7.41±0.07 | | 7.38±0.07 | | 7.34±0.10 | | 7.33±0.12 | | 0.078 | |  |
| Na (mmol/L) | 138.36±1.95 | 137.58±1.69 | | 134.52±2.50 | | 133.48±2.01 | | 136.45±2.66 | | 136.16±2.58 | | 137.39±2.23 | | 137.23±2.36 | | 138.33±2.24 | | 137.71±1.98 | | 138.91±2.13 | | 138.54±1.79 | | 0.460 | |  |
| K (mmol/L) | 3.94±0.34 | 3.91±0.29 | | 5.20±0.61 | | 5.42±0.84 | | 3.99±0.80 | | 3.99±0.91 | | 4.29±0.58 | | 4.51±1.40 | | 4.20±0.48 | | 4.28±0.46 | | 4.11±0.49 | | 4.13±0.47 | | 0.872 | |  |
| *Notes*: All times are approximate from the completion of the TBI, which is considered to be the onset of trauma (see Table 1). HCO_3_ = bicarbonate; PCO_2_ = partial pressure carbon dioxide; iCa = ionized calcium; pH = potential hydrogen; Na = sodium; K = potassium. Group × Time interaction *p* values are presented in the last column. | | | | | | | | | | | | | | | | | | | | | | | | | | |


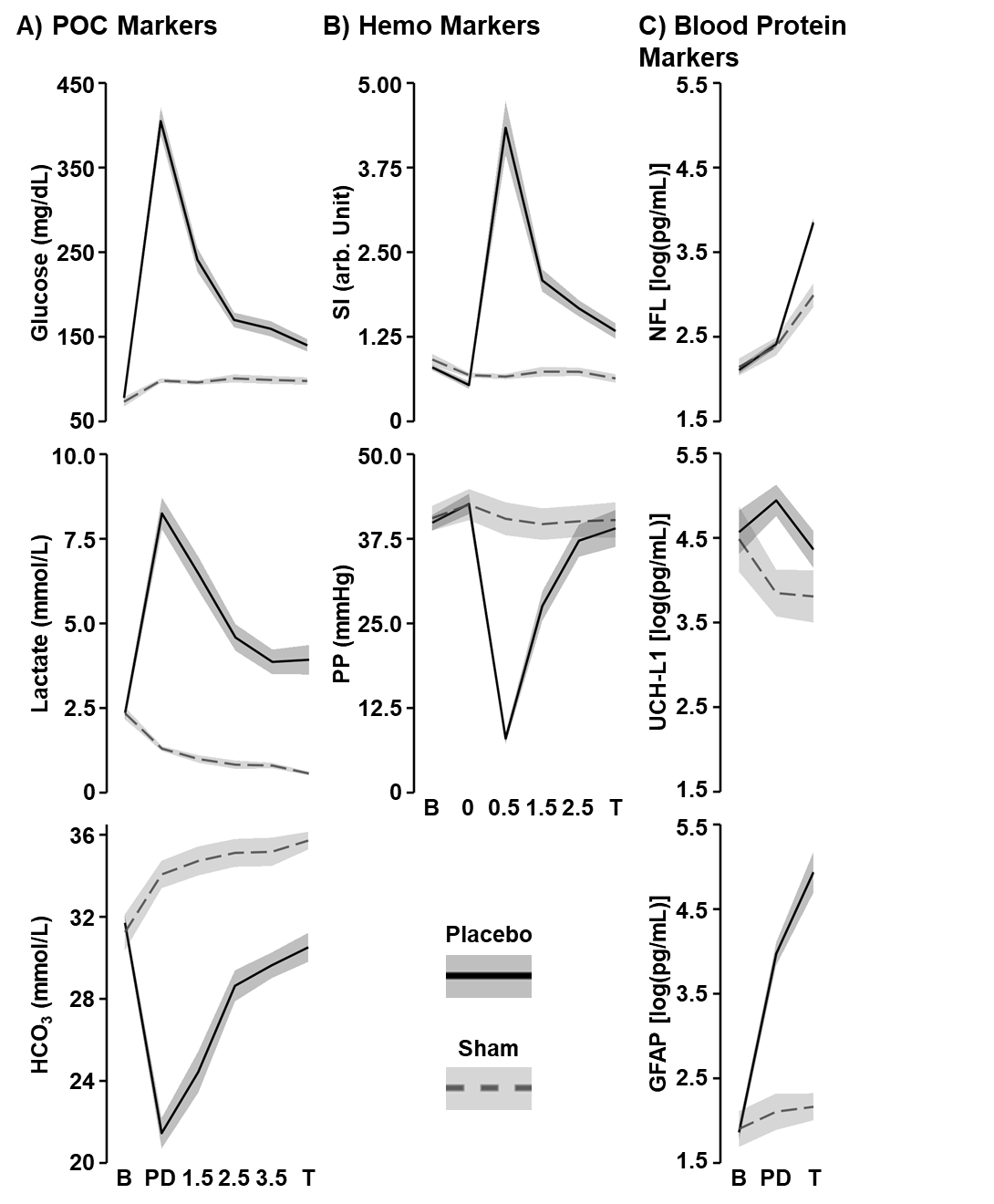


**Supplemental Figure 1:** Line plots for primary biomarkers to characterize model pathology in Placebo (N=34; solid line) versus Sham (N=12; dashed line) cohorts. Plotted data include primary point-of-care (POC) markers (Panel A; glucose, lactate and bicarbonate [HCO_3_]), primary invasive hemodynamic (Hemo) markers (Panel B; shock index [SI] and pulse pressure [PP]), and primary blood protein markers (Panel C; neurofilament light chain [NFL], *u*biquitin C-terminal hydrolase [UCH-L1] and glial fibrillary acidic protein [GFAP]). The mean and standard error are presented in each graph. Labels for the X-axis correspond to Table 1 from the main manuscript, but are presented in approximate hour units for Arabic numeral for display purposes. Potential time-point collections for the different biomarkers include baseline (B), immediately after traumatic brain injury (0), at 25 minutes corresponding with the end of blood loss (~0.5 hour), post-drug (PD), 85 minutes (~1.5 hours), 145 minutes (~2.5 hours) or 205 minutes (~3.5 hours) post-TBI, and at the terminal (T) experimental endpoint. Equivalent time points were sampled for the Sham cohort.


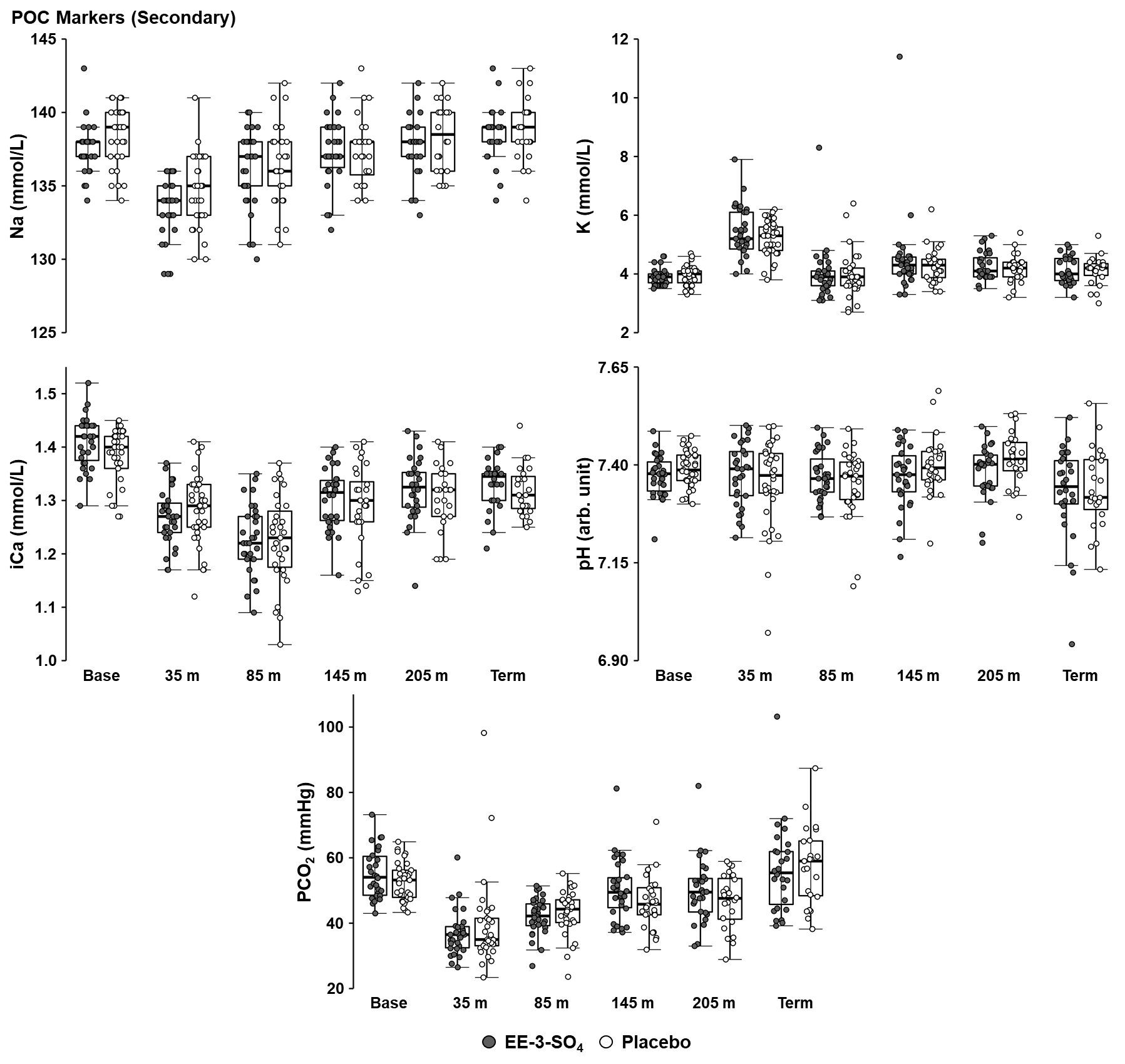


**Supplemental Figure 2:** Box-and-scatter plots depicting secondary point-of-care (POC) markers for EE-3-SO_4_ (filled circles) and Placebo (unfilled circles) cohorts. Collection of secondary POC markers (sodium [NA], potassium [K], ionized calcium [iCa], potential hydrogen [pH] and partial pressure of carbon dioxide [PCO_2_]) occurred at baseline (Base), following drug/placebo administration (35 m), at regular hour intervals post-trauma (85 m, 145 m, 205 m), and at the terminal experimental endpoint (Term; ~295 m post-traumatic brain injury). Times are given according to Table 1 in the main manuscript.


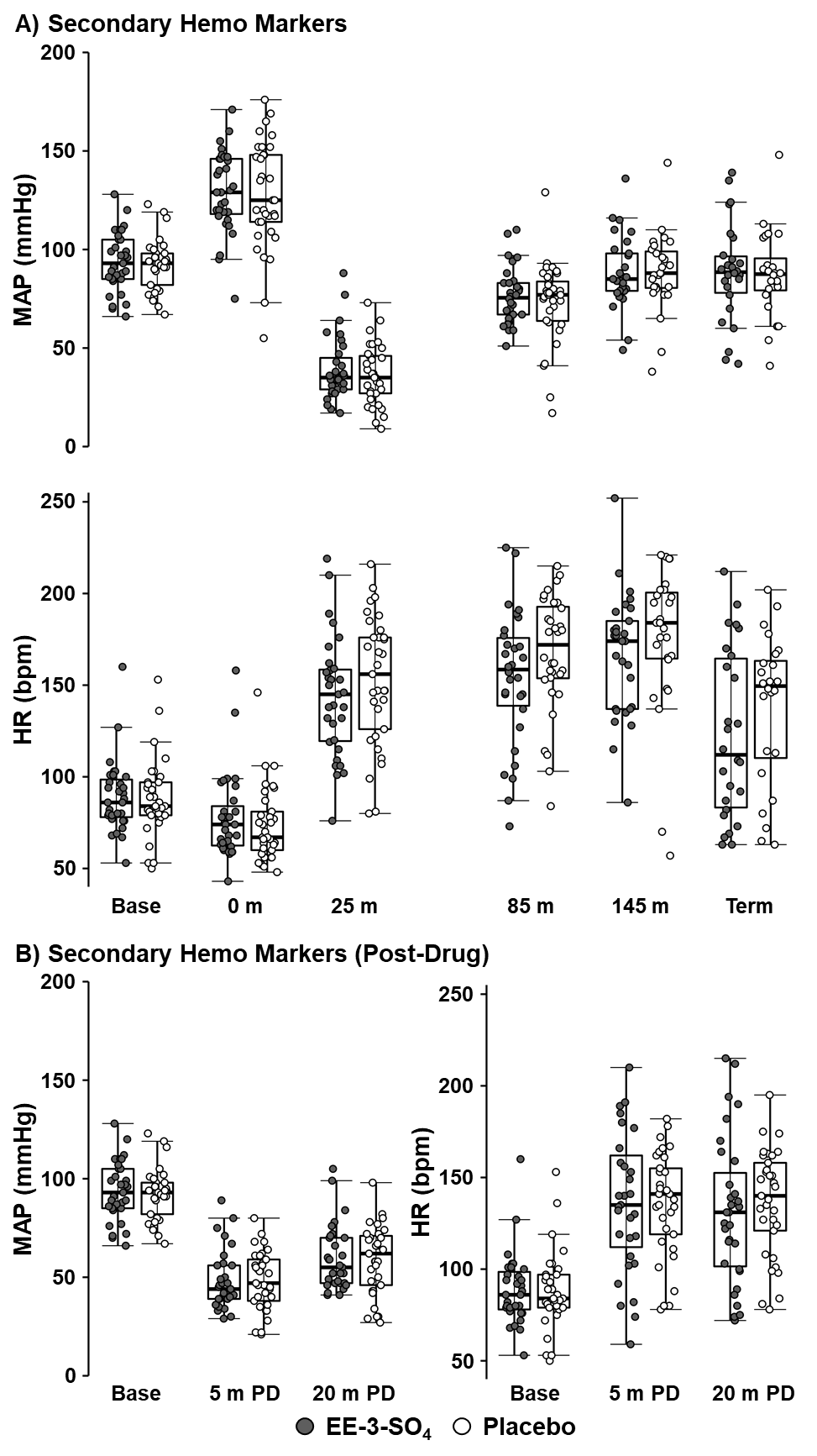


**Supplemental Figure 3:** Box-and-scatter plots for secondary invasive hemodynamic (Hemo) markers for EE-3-SO_4_ (filled circles) and Placebo (unfilled circles) cohorts. Secondary hemodynamic markers (heart rate [HR] and mean arterial pressure [MAP]) were continuously collected over the course of the entire experiment (Panel A), with data points displayed for baseline (Base), immediately post-traumatic brain injury (0 m), immediately post blood loss procedure (25 m), at hour intervals post-trauma (85 m and 145 m), and at the terminal experimental endpoint (Term). Panel B presents a smaller temporal window to capture the rapid effects (5 m and 20 m post-drug [PD]) of EE-3-SO_4_ administration. Times are given according to Table 1 in the main manuscript.


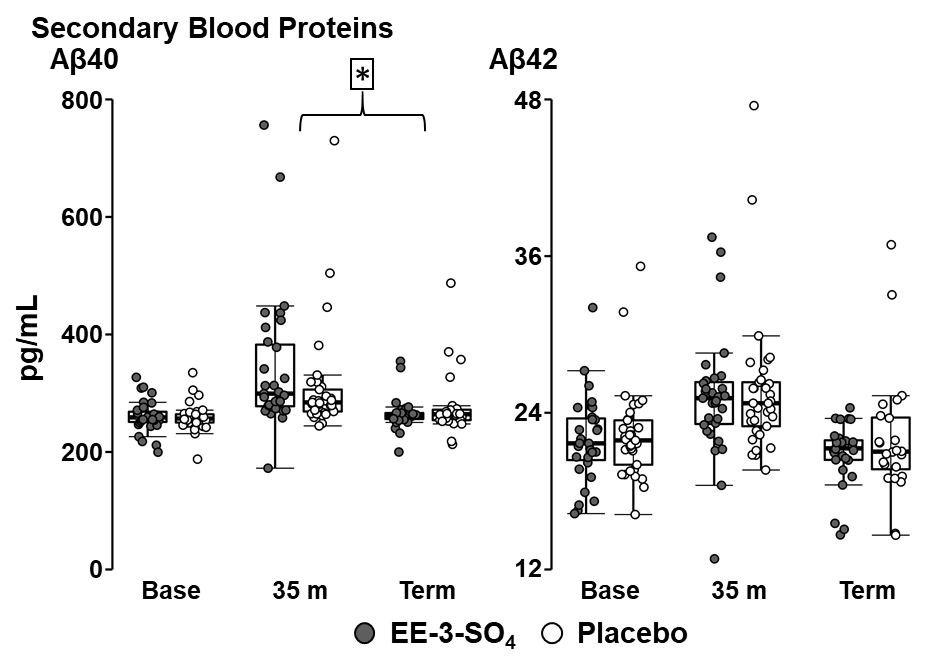


**Supplemental Figure 4:** Box-and-scatter plots for secondary blood biomarkers of amyloid beta 40 and 42 (Aβ40 and Aβ42) for EE-3-SO_4_ (filled circles) and Placebo (unfilled circles) cohorts. Blood biomarkers were collected at baseline (Base), immediately post-drug (35 m) and at the terminal experimental endpoint (Term; ~295 m post-traumatic brain injury). Aβ40 was associated with a significant Group × Time interaction, with an asterisk denoting a larger change from post-drug to Term time points in the EE-3-SO_4_ cohort. Graphed Aβ data were corrected for batch effects (batch 2 – [mean difference between baseline batch 2 – batch 1 results]).
